# The RhoG-Binding Domain of ELMO1 Rescues the PTENopathy-like Phenotype in Oligodendroglial FBD-102b Cells

**DOI:** 10.3390/ijms27083457

**Published:** 2026-04-12

**Authors:** Mikito Takahashi, Mei Tanaka, Hideji Yako, Yuki Miyamoto, Junji Yamauchi

**Affiliations:** 1 Laboratory of Molecular Neuroscience and Neurology, Tokyo University of Pharmacy and Life Sciences, Hachioji, Tokyo 192-0392, Japanhyako@toyaku.ac.jp (H.Y.); miyamoto-y@ncchd.go.jp (Y.M.); 2 Department of Pharmacology, National Research Institute for Child Health and Development, Setagaya, Tokyo 157-8535, Japan; 3 Department of Biological Science, Tokyo College of Biotechnology, Oota, Tokyo 144-0032, Japan

**Keywords:** PTEN, RBD, Akt, oligodendrocyte, differentiation

## Abstract

Oligodendroglial cells are the myelinating glial cells of the central nervous system (CNS), and their morphological differentiation is a prerequisite for efficient myelin formation, which is essential for proper neuronal function. While oligodendroglial morphological changes normally proceed through tightly regulated developmental transitions, disruption of the underlying molecular mechanisms can lead to aberrant cellular phenotypes characterized by either premature, insufficient, or excessive differentiation. Although the phosphatidylinositol 3-kinase (PI3K) and its downstream Akt kinase signaling are well established as major drivers of oligodendrocyte morphological differentiation, myelination, and CNS white matter formation, how its negative regulator, phosphatase and tensin homolog (PTEN), is involved in the regulation of oligodendroglial morphogenesis remains incompletely understood. Recent genetic studies have highlighted a spectrum of disorders caused by PTEN dysfunction, conceptually established but currently evolving as PTENopathy, which has been partially associated with white matter abnormalities. Here, we report that, in an experimental model using the FBD-102b cell line, a well-established model of oligodendroglial cell differentiation, chemical inhibition of PTEN enhances pronounced morphological changes characterized by widespread membranes, accompanied by increased expression of differentiation and/or myelin marker proteins. We then focused on Rho family small GTPases, central regulators of cell morphogenesis, and examined their potential involvement downstream of this signaling. Expression of the RhoG-binding domain (RBD) of engulfment and cell motility 1 (ELMO1) attenuated the increased morphological changes. Similarly, inhibition of downstream Akt signaling also reversed these changes. Taken together, these results provide insight into how balanced regulation between PTEN and downstream signaling molecules governs oligodendroglial cell differentiation and suggest that dysregulation of this signaling equilibrium may contribute to cellular phenotypes relevant to disease-associated cellular alterations.

## 1. Introduction

Oligodendroglial cells (also called oligodendrocytes) in the central nervous system (CNS) are the principal glial cells responsible for myelinating neuronal axons through their differentiated plasma membranes, which is essential for maintaining conduction velocity and supporting various neuronal functions [1,2]. Oligodendrogenesis and subsequent myelination proceed through a highly orchestrated series of developmental steps, including initial steps such as proliferation and migration, mid-stage steps such as fate determination and morphological differentiation, and late steps such as myelin protein synthesis, axonal myelination, and white matter formation [3,4]. These steps are tightly regulated by multiple intracellular and intercellular signaling pathways, including growth factor receptors and serine/threonine kinases [3,4,5,6,7,8,9,10]. Disruption of the molecular mechanisms that govern oligodendroglial morphological changes has been implicated in a wide range of white matter disorders, including hereditary demyelinating diseases and neurodevelopmental or neurodegenerative conditions. Therefore, elucidating how these cellular events are dysregulated, including whether morphological remodeling is premature, insufficient, or excessive, is critical for possible development of effective therapeutic strategies [11,12,13,14,15,16,17,18].

Among the regulatory pathways controlling oligodendroglial cell morphogenesis, the phosphatidylinositol 3-kinase (PI3K) and Akt kinase signaling axis plays a critical role in coordinating oligodendroglial cell morphological differentiation and, consequently, myelination [5,6]. In contrast, phosphatase and tensin homolog deleted from chromosome 10 (PTEN), which is a well-known cell growth and tumor suppressor, functions as a negative regulator of the PI3K and Akt signaling pathway by dephosphorylating phosphatidylinositol 3,4,5-trisphosphate. In the CNS, PI3K and PTEN exert pleiotropic effects, influencing axon and dendrite development and synaptic plasticity in neurons, while in the oligodendroglial cell lineage, fluctuations in PI3K and PTEN activities affect cell proliferation, differentiation competence, and myelin synthesis capacity [19,20].

The term “PTENopathy”, while a long-standing clinical concept, is still evolving and is likely to become an increasingly important conceptual entity. It refers to a spectrum of disorders caused by functional loss or dysregulation of PTEN, leading to activation of PI3K and its well-known downstream Akt signaling cascade [19,20]. In the white matter of the CNS, an imbalance of PTEN activity in certain conditions, including autism spectrum disorder (ASD) [21,22,23], Cowden syndrome [24], and macrocephaly [24], has been partially associated with abnormalities in mature myelin architecture. Aberrantly increased downstream signaling has been reported to be associated with enhanced oligodendroglial cell differentiation and myelination, characterized by premature and/or excessive differentiation [25].

Mouse model studies have demonstrated that loss or inhibition of PTEN promotes abnormal oligodendroglial cell-derived white matter expansion in the brain, contributing to pathologies such as ASD [26,27]. Specifically, genetic deletion or pharmacological inhibition of PTEN in oligodendroglial lineage cells leads to excessive myelin membrane outgrowth and aberrant white matter enlargement in vivo. In contrast, proper differentiation and functional myelination of oligodendroglial cells depend on tightly controlled PTEN signaling, as disruption of PTEN impairs myelin and axonal integrity without promoting effective remyelination [28,29]. Accordingly, PTEN dysfunction has come to be recognized as one of the key molecular drivers of white matter pathology and contrasts with the features observed in hypomyelinating conditions [4,7], where insufficient myelin formation rather than excessive membrane expansion is observed.

Small GTPases of the Rho family are key molecular switches that regulate a wide range of cellular events, including cell morphogenesis [30,31]. They function by cycling between an active GTP-bound state and an inactive GDP-bound state, effectively transmitting upstream signals to downstream effectors to drive developmental processes [30,31,32,33]. Members of the Rho family, including RhoA, Rac1, and Cdc42, have been extensively studied and play distinct yet overlapping roles in organizing signaling networks that precisely control cell morphogenesis [30,31,32,33]. Among them, Rac1 possesses a particularly strong ability to induce membrane ruffling at the cell periphery, a critical step in expanding cellular boundaries [31,32,33,34]. Notably, RhoG acts upstream of Rac1, illustrating its unique position in the hierarchical regulation of Rho GTPases [35,36,37]. Given their fundamental roles in promoting neural maturation, dysregulation of signaling through Rho GTPases has been implicated in pathological conditions, including neurodevelopmental disorders [30,37,38,39].

Despite these insights regarding PTEN, the mechanisms by which PTEN inhibition affects the differentiation program in oligodendroglial cells remain largely unknown. The purpose of this study is to determine whether a commonly employed chemical inhibitor of PTEN [40,41,42,43] promotes morphological differentiation in FBD-102b cells, a widely used model of oligodendroglial cell differentiation [44,45,46,47]. Furthermore, we explore how this phenotype can be modulated at the molecular and cellular levels. Through this approach, we aim to provide insight into PTEN inhibition-associated cellular mechanisms in an in vitro context and to highlight potential disease-specific drug target(s) that could inform future strategies.

## 2. Results

### 2.1. Inhibition of PTEN Increases Cell Morphological Changes

**Figure 1:**
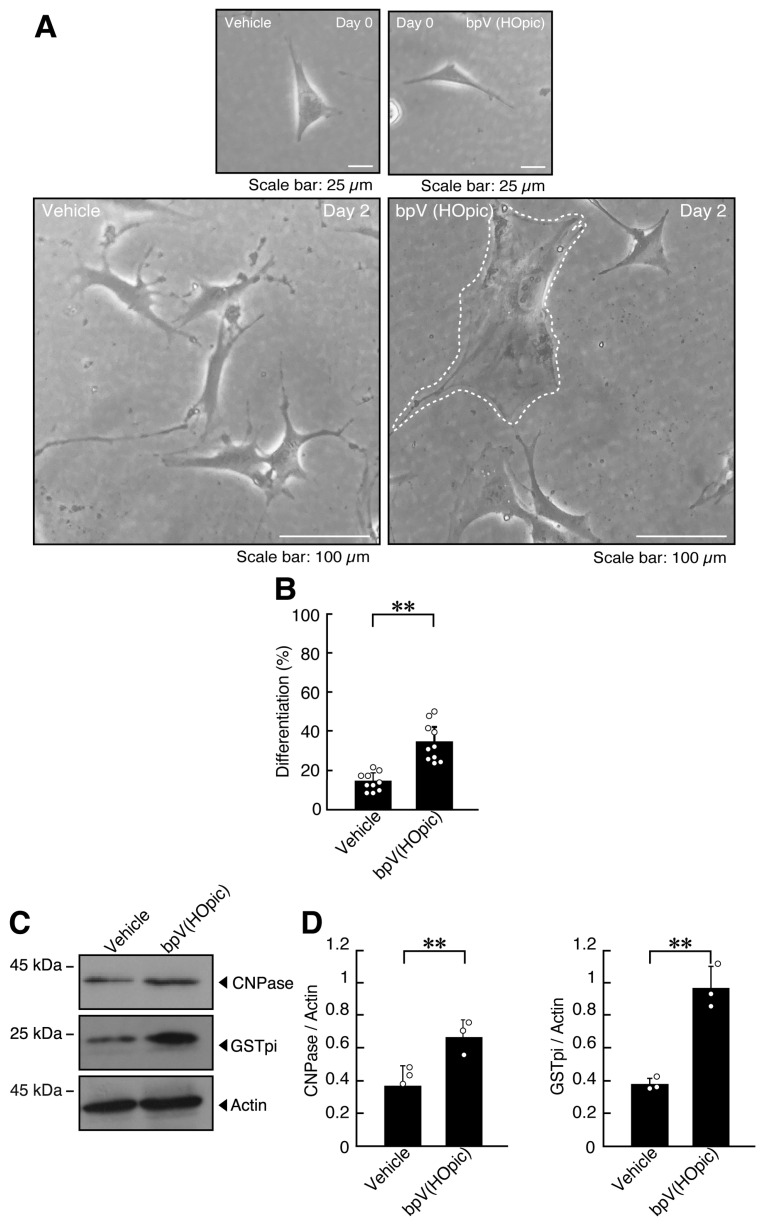
Treatment with bpV(HOpic), a PTEN inhibitor, increases excessive morphological differentiation. (**A**,**B**) FBD-102b cells were differentiated for 2 days in the presence or absence (vehicle control) of 5 micromolar of the PTEN inhibitor bpV(HOpic). Representative images are shown, and the percentages of differentiated cells are quantified (**, *p* < 0.01; *n* = 10 fields). Values: Individual values are plotted as open circles in a graph. The representative differentiated cell is outlined by a white dotted line. (**C**,**D**) Cell lysates prepared 2 days after induction of differentiation were subjected to immunoblotting using antibodies against GSTpi, CNPase, and control actin proteins. Quantifications of each immunoreactive band intensity normalized to the actin loading control are shown (**, *p* < 0.01; *n* = 3 blots). Values: Individual values are plotted as open circles.

To model PTEN deficiency, including loss or impaired activity, we treated oligodendroglial FBD-102b cells with bpV(HOpic), a specific inhibitor of PTEN [41,42]. After 2 days of differentiation, bpV(HOpic)-treated cells exhibited an approximately twofold increase in the proportion of cells with widespread membranes compared with vehicle-treated controls (Figure 1A,B and Figure S1).

Forty of the cells exhibited differentiated phenotypes after 2 days, and this proportion did not increase further by day 6. In contrast, the control group displayed less than 20% differentiated phenotypes at 2 days, with only a modest increase to approximately 30% at days 4 and 6. To further investigate the effects of these two PTEN inhibitors, we examined the phosphorylation status of key signaling molecules in the immediate downstream pathway of PTEN, specifically mTOR kinase at its autophosphorylation site (Ser-1261) and Akt at its activation site (Thr-308). Both inhibitors resulted in increasing the phosphorylation levels of these proteins, indicating effective suppression of PTEN activity at the inhibitor concentrations used in these experiments (Figure S2). In addition, quantitative analyses confirmed a significant increase in the cell area of cells with widespread membranes compared with vehicle-treated controls (Figure S3).

Markers of oligodendroglial differentiation and myelination, including glutathione S-transferase (GST) pi and CNPase, were also increased in bpV(HOpic)-treated cells (Figure 1C,D). Similarly, other markers of differentiation and myelination, proteolipid protein 1 (PLP1) and claudin-11 (CLDN11), were increased in bpV(HOpic)-treated cells (Figure S4). These results indicate that inhibition of PTEN by bpV(HOpic) is associated with increased morphological differentiation.

**Figure 2:**
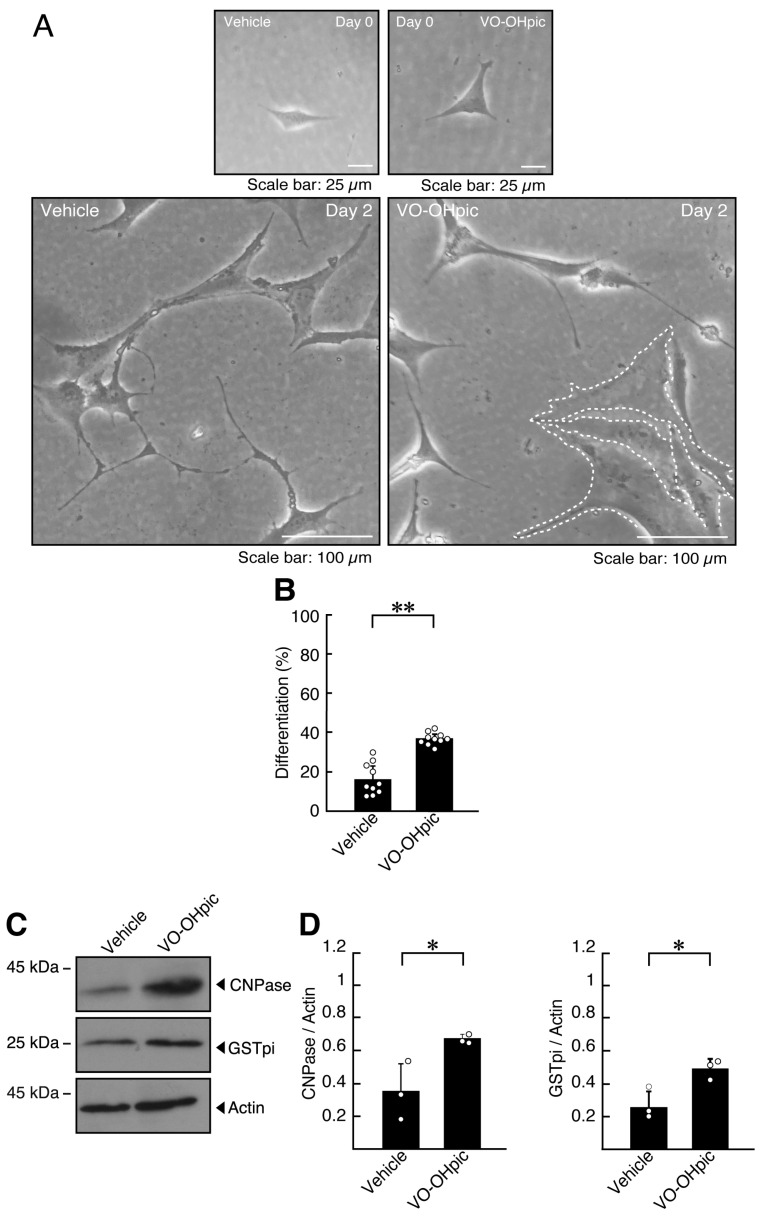
Treatment with VO-OHpic, an additional PTEN inhibitor, increases excessive morphological differentiation. (**A**,**B**) FBD-102b cells were differentiated for 2 days in the presence or absence (vehicle control) of 3 micromolar of VO-OHpic. Representative images are shown, and the percentages of differentiated cells are quantified (**, *p* < 0.01; *n* = 10 fields). Values: Individual values are plotted as open circles in a graph. The representative differentiated cell is outlined by a white dotted line. (**C**,**D**) Cell lysates prepared 2 days after induction of differentiation were subjected to immunoblotting using antibodies against GSTpi, CNPase, and control actin proteins. Quantifications of each immunoreactive band intensity normalized to the actin loading control are shown (*, *p* < 0.05; *n* = 3 blots). Values: Individual values are plotted as open circles.

We next examined the effects of VO-OHpic, another PTEN inhibitor [43]. Consistent with the results of bpV(HOpic), VO-OHpic-treated cells exhibited a twofold increase in the frequency of cells with widespread membranes compared to vehicle-treated controls (Figure 2A,B, Figures S1 and S3), along with increased expression of markers GSTpi and CNPase (Figure 2C,D and Figure S4).

Taken together, these results indicate that PTEN inhibition by either compound is associated with membrane expansion and increased levels of differentiation markers in oligodendroglial cells.

### 2.2. RhoG-Binding Domain Attenuates Increased Morphological Changes

We next sought to attenuate, or potentially reverse, the morphological alterations induced by PTEN inhibition. To this end, we tested the effect of the GTP-bound RhoG (active RhoG)-binding domain (RBD) of engulfment and cell motility 1 (ELMO1) on cells. RBD inhibits the small GTPase Rac1 signaling cascade, which controls dynamic cell morphogenesis [36,37]. Rac1 generally acts downstream of a genetically conserved core Rac1 regulatory signaling module composed of ELMO and dedicator of cytokinesis protein (DOCK) molecules [35,36].

**Figure 3:**
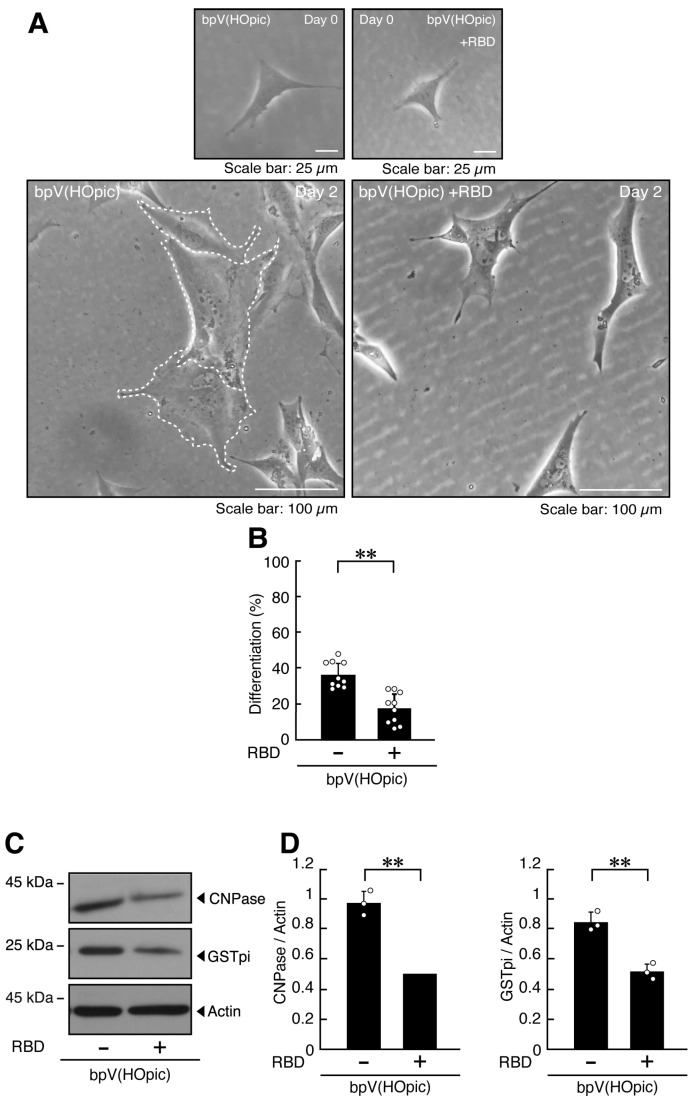
RBD recovers the cellular phenotype elicited by bpV(HOpic). (**A**,**B**) FBD-102b cells were transfected with or without (control vector) the plasmid encoding RBD and were differentiated for 2 days in the presence of bpV(HOpic). Representative images are shown, and the percentages of differentiated cells are quantified (**, *p* < 0.01; *n* = 10 fields). Values: Individual values are plotted as open circles in a graph. The representative differentiated cell is outlined by a white dotted line. (**C**,**D**) Cell lysates prepared 2 days after induction of differentiation were subjected to immunoblotting using antibodies against GSTpi, CNPase, and control actin proteins. Quantifications of each immunoreactive band intensity normalized to the actin loading control are shown (**, *p* < 0.01; *n* = 3 blots). Values: Individual values are plotted as open circles.

Transfection with RBD attenuated the phenotypes of bpV(HOpic)-treated cells, reducing the proportion of cells with widespread membranes by approximately half (Figure 3A,B and Figure S3). Similarly, transfection with RBD decreased the expression levels of GSTpi, CNPase, PLP1, and CLDN11 (Figure 3C,D and Figure S5).

**Figure 4:**
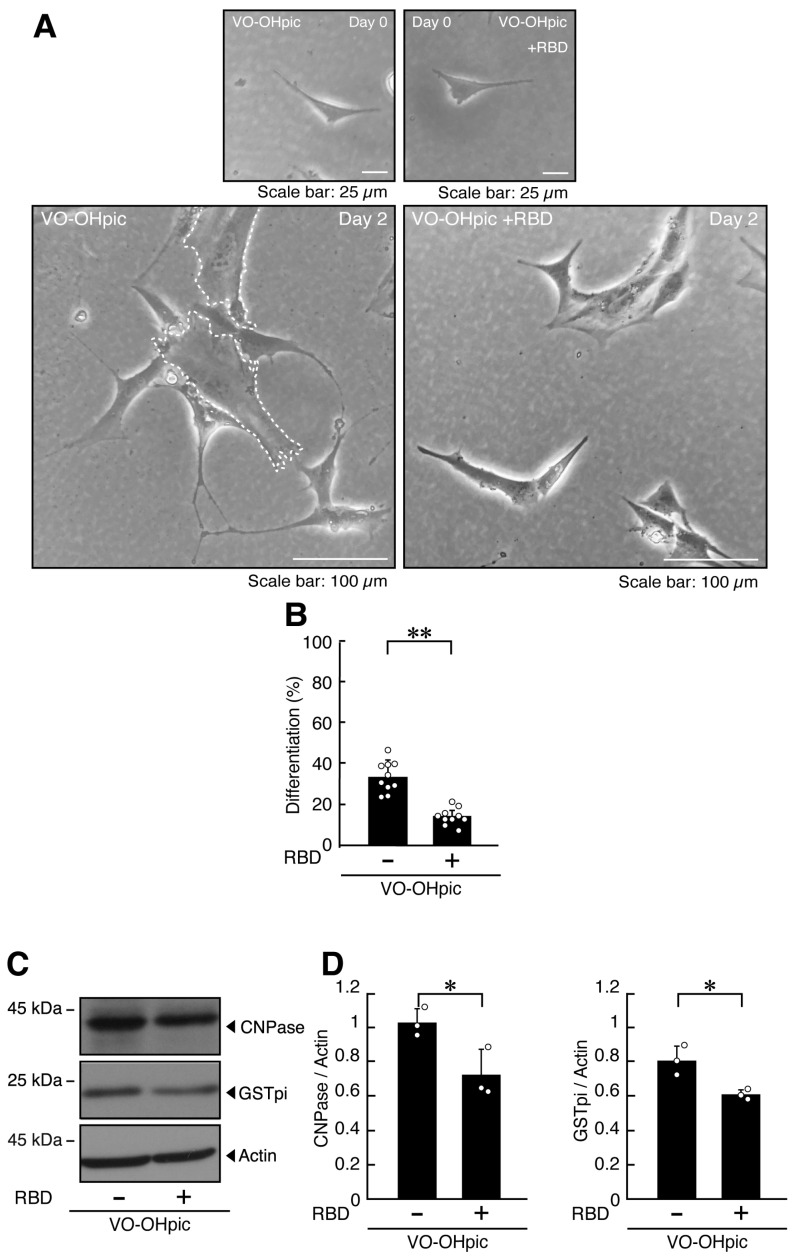
RBD recovers the cellular phenotype elicited by VO-OHpic. (**A**,**B**) FBD-102b cells were transfected with or without (control vector) the plasmid encoding RBD and were differentiated for 2 days in the presence of VO-OHpic. Representative images are shown, and the percentages of differentiated cells are quantified (**, *p* < 0.01; *n* = 10 fields). Values: Individual values are plotted as open circles in a graph. The representative differentiated cell is outlined by a white dotted line. (**C**,**D**) Cell lysates prepared 2 days after induction of differentiation were subjected to immunoblotting using antibodies against GSTpi, CNPase, and control actin proteins. Quantifications of each immunoreactive band intensity normalized to the actin loading control are shown (*, *p* < 0.05; *n* = 3 blots). Values: Individual values are plotted as open circles.

The effects of VO-OHpic were likewise recovered by RBD (Figure 4A–D, Figures S3 and S5), indicating that RBD can counteract, at least in part, both the morphological and molecular consequences of PTEN inhibition.

Building on these findings, we next examined whether PTEN inhibition is associated with changes in RhoG activity. Using an affinity-precipitation assay using RBD, we observed an increase in RhoG activity in cells treated with either bpV(HOpic) or VO-OHpic (Figure S6). These results suggest that PTEN inhibition promotes RhoG activation and, together with the effects of RBD expression, are consistent with the involvement of signaling through RhoG in the observed phenotypic changes.

### 2.3. Inhibition of Akt Kinase Attenuates Morphological Changes

We next explored whether the morphological changes induced by PTEN inhibition could be attenuated or reversed by modulation of the downstream pathway through RhoG. To this end, we evaluated the effects of curcumin, an inhibitor of the Akt signaling pathway [48,49,50,51], on cells, since the PI3K and Akt signaling unit is one of the major Rac1 molecular targets [52,53,54,55] and plays a central role in enhancing oligodendroglial differentiation and myelination [3,4].

**Figure 5:**
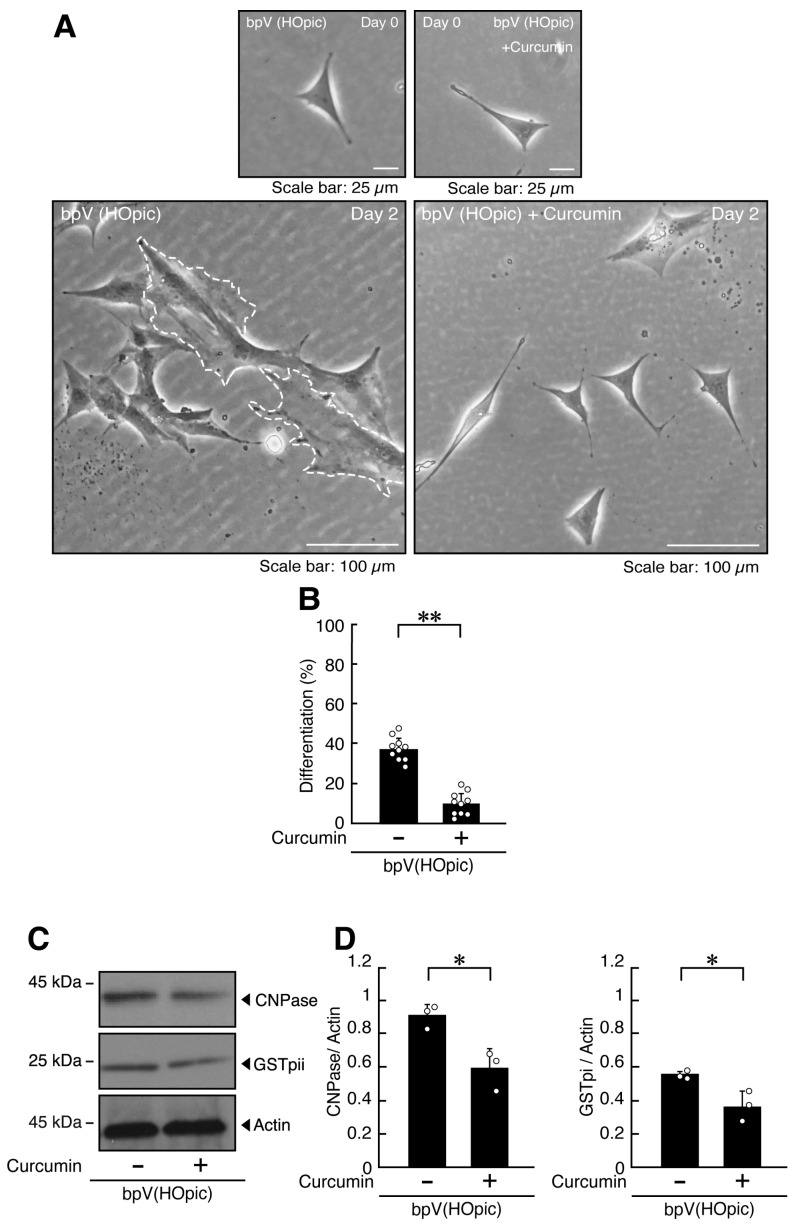
Curcumin recovers the cellular phenotype elicited by bpV(HOpic). (**A**,**B**) FBD-102b cells were differentiated for 2 days in the presence of bpV(HOpic), with or without (vehicle control) 10 micromolar of curcumin. Representative images are shown, and the percentages of differentiated cells are quantified (**, *p* < 0.01; *n* = 10 fields). Values: Individual values are plotted as open circles in a graph. The representative differentiated cell is outlined by a white dotted line. (**C**,**D**) Cell lysates prepared 2 days after induction of differentiation were subjected to immunoblotting using antibodies against GSTpi, CNPase, and control actin proteins. Quantifications of each immunoreactive band intensity normalized to the actin loading control are shown (*, *p* < 0.05; *n* = 3 blots). Values: Individual values are plotted as open circles.

Curcumin treatment substantially attenuated the cellular morphology elicited by bpV(HOpic), reducing the proportion of cells with widespread membranes to approximately 50% (Figure 5A,B and Figure S3). Consistent with these morphological changes, curcumin treatment was associated with decreased expression levels of the markers GSTpi, CNPase, PLP1, and CLDN11 (Figure 5C,D and Figure S7).

Also, treatment with MK-2206, a highly specific Akt inhibitor [56,57], significantly attenuated these effects (Figure S8). Notably, the reduction was more profound than anticipated. This pronounced inhibition may be attributed to the fact that a certain level of Akt signaling is fundamentally required for the differentiation mechanism itself [3,4].

**Figure 6:**
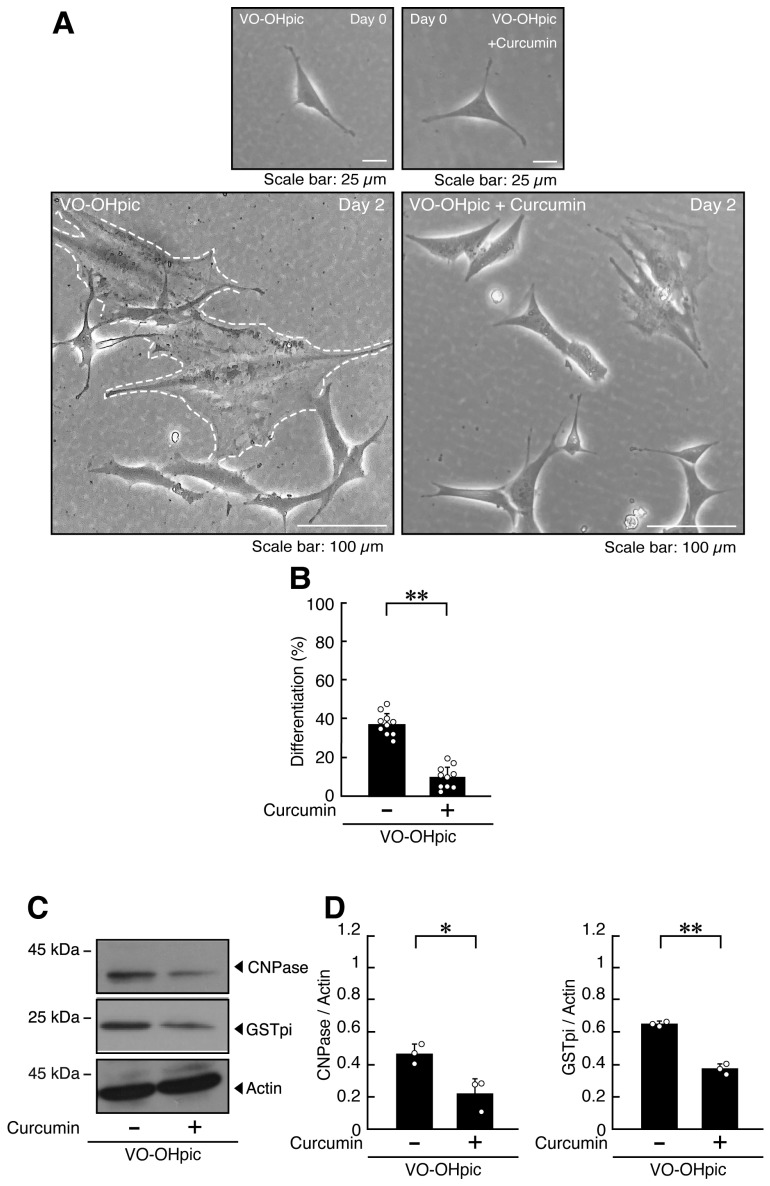
Curcumin recovers the cellular phenotype elicited by VO-OHpic. (**A**,**B**) FBD-102b cells were differentiated for 2 days in the presence of VO-OHpic, with or without (vehicle control) 10 micromolar of curcumin. Representative images are shown, and the percentages of differentiated cells are quantified (**, *p* < 0.01; *n* = 10 fields). Values: Individual values are plotted as open circles in a graph. The representative differentiated cell is outlined by a white dotted linen a graph. (**C**,**D**) Cell lysates prepared 2 days after induction of differentiation were subjected to immunoblotting using antibodies against GSTpi, CNPase, and control actin proteins. Quantifications of each immunoreactive band intensity normalized to the actin loading control are shown (**, *p* < 0.01, *, *p* < 0.05; *n* = 3 blots). Values: Individual values are plotted as open circles.

Similar ameliorative effects of curcumin were observed in cells treated with VO-OHpic (Figure 6A–D, Figures S3 and S7), suggesting that curcumin can alleviate, at least in part, both the morphological and molecular alterations resulting from PTEN inhibition.

### 2.4. Transfection of RBD or Inhibition of Akt Kinase Attenuates Akt Phosphorylation

**Figure 7:**
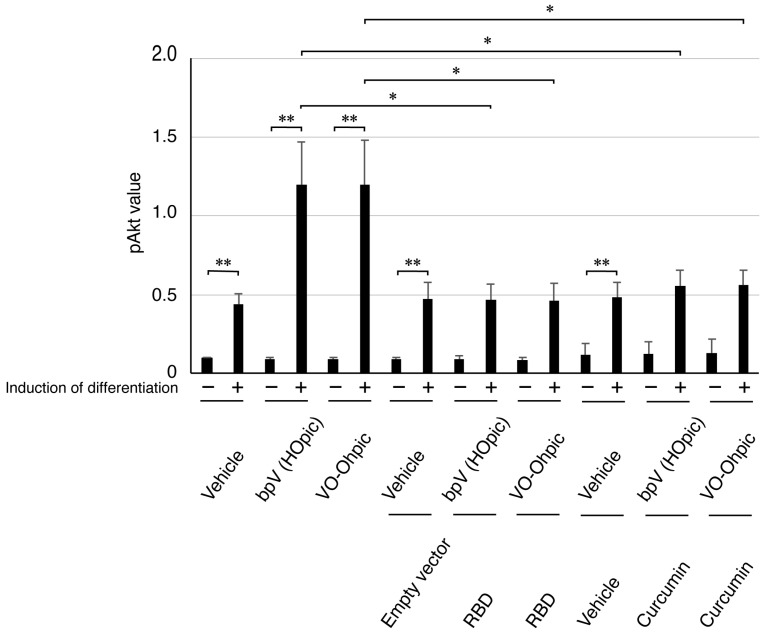
RBD or curcumin restores bpV(HOpic)- or VO-OHpic-altered phosphorylated Akt levels. Lysates from FBD-102b cells transfected with the plasmid encoding RBD or treated with curcumin, in the presence or absence of bpV(HOpic), were subjected to a commercially available assay kit to measure phosphorylated Akt levels. The numerical values were quantified and plotted in the accompanying graph (**, *p* < 0.01 and *, *p* < 0.05; *n* = 3). Additionally, lysates from FBD-102b cells transfected with the plasmid encoding RBD or treated with curcumin, in the presence or absence of VO-OHpic, were subjected to a commercially available assay kit to measure phosphorylated Akt levels. The numerical values were quantified and plotted in the accompanying graph (**, *p* < 0.01 and *, *p* < 0.05; *n* = 3).

To further elucidate the related mechanism, we investigated whether the transfection of RBD or the inhibition of Akt signaling modulates the Akt phosphorylation induced by bpV(HOpic) or VO-OHpic. Focusing on Ser-473, which functions as an autophosphorylation site [42,43], we observed that both RBD transfection and Akt inhibition effectively suppressed the phosphorylation triggered by these compounds (Figure 7). These results provide evidence that the precise modulation of Akt signaling pathways plays a crucial role in regulating morphological differentiation.

## 3. Discussion

The primary objective of this study was to develop a possible in vitro model of PTEN inhibition, an emerging conceptual entity, with a focus on investigating the effects of PTEN on oligodendroglial cell morphological differentiation using the FBD-102b cell line, a widely utilized model of oligodendroglial differentiation. We demonstrate that chemical inhibition of PTEN induces pronounced morphological changes, characterized by widespread membrane expansion and accompanied by increased expression of differentiation- and/or myelination-associated marker proteins. These observations suggest that PTEN signaling functions, at least in part, as a negative regulator of oligodendroglial cell morphogenesis. Importantly, PTEN inhibition appears to bias oligodendroglial cells toward an extensive differentiation-like state, although whether this state fully recapitulates physiological maturation or in vivo myelination remains uncertain.

White matter abnormalities observed in ASD, Cowden syndrome, and macrocephaly often suggest that these conditions are associated with enhanced oligodendrocyte differentiation and expanded white matter [21,22,23,24,25]. Although ASD is a genetically heterogeneous disorder, loss-of-function mutations in PTEN constitute an established genetic cause of a subset of ASD cases, particularly those associated with macrocephaly [21,22,23,24]. Cowden syndrome is primarily caused by germline loss-of-function mutations in PTEN, leading to dysregulated signaling, including through the Akt signaling pathway [21,22,23,24]. Accordingly, Cowden syndrome is associated with neurodevelopmental phenotypes characterized by ASD-like features and macrocephaly. Activation of signaling pathways downstream of PTEN often promotes excessive oligodendroglial differentiation and subsequent myelination [26,27,28,29]. In this context, our experimental model may provide a useful platform to dissect both shared and distinct molecular mechanisms underlying aberrant PTEN-related signaling in these neurodevelopmental conditions.

Accumulating evidence has established that activation of intracellular signaling pathways, including the Akt pathway, promotes oligodendrocyte lineage progression, differentiation, and myelination in both in vitro and in vivo contexts [5,6]. In contrast, comparatively less attention has been devoted to understanding how PTEN-mediated negative regulation contributes to the fine control of morphological changes underlying developmental maturation and subsequent myelination [26,27,28,29]. Our results support the idea that loss or reduction of PTEN activity disrupts the balance of intracellular signaling states, leading to excessive activation of downstream pathways and, consequently, cellular phenotypes indicative of potentially abnormal or dysregulated morphological outcomes. Collectively, PTEN is thought to act as a quantitative regulator that constrains signaling amplitude [5,6,28,29], thereby ensuring appropriate and orderly oligodendroglial morphological progression prior to the onset of myelination.

We explored the potential involvement of Rho family small GTPase pathways, which are well known to regulate actin cytoskeletal dynamics, membrane remodeling, and cellular polarity, in this study [38,39]. These activities are critical for subsequent changes in oligodendroglial cells, including morphological differentiation and, ultimately, myelination [5,6]. Notably, expression of RBD attenuated the morphological changes induced by PTEN inhibition, indicating that RhoG-dependent signaling contributes, at least in part, to the observed phenotypes. RhoG (*Caenorhabditis elegans* MIG-2 homologue) acts upstream of a genetically conserved signaling module that couples the mammalian ELMO (*C. elegans* CED-12 homologue) and mammalian DOCK (*C. elegans* CED-5 homologue) complex to activate Rac1 (*C. elegans* CED-10 homologue) to JNK (*C. elegans* JNK-1 homologue) [35]. This pathway itself is known to regulate dynamic cell morphogenesis. In addition, because Rac1 is a central regulator of membrane ruffling and lamellipodia formation [38,39], it is plausible that oligodendroglial membrane expansion may be aberrantly stimulated downstream of PTEN inhibition via Rac1 signaling.

The present findings may provide mechanistic insight into cellular aspects of disorders associated with PTEN dysfunction. Increasing evidence suggests that oligodendroglial cells in these conditions can exhibit altered, and in some cases excessive or ectopic, myelination. In this context, our data are consistent with the possibility that reduced PTEN signaling activity biases oligodendroglial cells toward excessive morphological differentiation. Importantly, such exaggerated morphological changes may not necessarily correspond to optimal or properly organized oligodendroglial cell differentiation. Taken together, these observations raise the possibility that both reduced and increased PTEN pathway activities could be detrimental, highlighting the importance of maintaining an appropriate signaling balance. Further studies employing genetic and in vivo models can be required to clarify how alterations in PTEN-regulated morphological differentiation impact oligodendroglial cell developmental integrity and mature neural circuitry.

Several limitations of the present study should be acknowledged. First, our analyses were performed using a cell line-based system, which may not fully recapitulate the complex cellular interactions and temporal regulation present in the intact CNS. Second, chemical inhibition of PTEN may have off-target effects that cannot be completely excluded, although two different inhibitors were utilized in this study. Third, although FBD-102b cells represent a robust and well-established model of oligodendroglial differentiation, they do not necessarily reflect human oligodendrocytes, which limits translational relevance. Future studies using human iPSC-derived oligodendrocytes and/or in vivo models, including genetically modified mice, are needed to address these limitations.

## 4. Materials and Methods

### 4.1. Key Materials

The key antibodies and plasmids used in this study are listed in Table 1.

### 4.2. Cell Culture

The FBD-102b cell line (Cell ID: RCB4965; Riken, Saitama, Japan), a mouse oligodendroglial precursor cell line, was cultured on cell and tissue culture dishes (Nunc brand, ThermoFisher Scientific, Waltham, MA, USA) in Dulbecco’s modified Eagle medium/F-12 medium (DMEM/F-12, Fujifilm, Tokyo, Japan; Nacalai Tesque, Kyoto, Japan) supplemented with 10% heat-inactivated fetal bovine serum (FBS) and penicillin-streptomycin antibiotic mixture (PenStrep; ThermoFisher Scientific or Nacalai Tesque) in 5% CO_2_ at 37 °C. To induce differentiation, cells were plated on culture dishes coated with polylysine (Nacalai Tesque), which provides a highly positive charge. Differentiation was induced by culturing the cells in the presence or absence of 5 micromolar of bpV(Hopic) 3 micromolar of VO-OHpic, 10 micromolar of curcumin, and/or 1 micromolar of MK-2206 in medium containing 0.5% FBS for several days in 5% CO_2_ at 37 °C. The experimental concentrations were optimized by considering their cellular effects, with reference to the values reported in the ChEMBL database (https://www.ebi.ac.uk/chembl/, accessed on 1 August 2025). Cells whose processes extended sufficiently to occupy an area equivalent to or larger than a 25-micrometer-diameter circle were generally classified as exhibiting differentiated phenotypes [44,45,46,47]. All image analyses were performed in a blinded manner. Under these conditions, trypan blue (Nacalai Tesque)-positive attached cells accounted for less than 2.5% of the population [45,46]. In addition, we have confirmed the effects of these two PTEN inhibitors at the concentrations used in experiments. The phosphorylation status of signaling molecules in the immediate downstream pathway of PTEN, mTOR at its autophosphorylation site (Ser-1261) and Akt at its activation site (Thr-308) were apparently increased (see Figure S2).

Cell morphologies were visualized using a microscopy system equipped with the i-NTER LENS set (Micronet, Inc., Saitama, Japan) and i-NTER SHOT software (ver. 2, Micronet, Inc.). Multiple samples were used for analyzing cells large enough to accommodate a 25-micrometer-diameter circle or cell areas themselves with Fiji software (ver. Java 8, downloaded from https://imagej.nih.gov/, accessed on 1 August 2025). For imaging analysis, unless otherwise indicated, ten microscopic fields were randomly selected from ten independent dishes. The data were obtained from five independent experiments (biological replicates), each performed using independently prepared cell cultures. All images were acquired using identical imaging settings across experimental groups.

### 4.3. Transfection

Cells were transfected with the respective plasmids using the HilyMax transfection kit (Dojindo, Kumamoto, Japan) in accordance with the manufacturer’s instructions. The medium was replaced 4 h post-transfection. Cells were typically used for biochemical experiments more than 48 h post-transfection [37].

### 4.4. Cell Lysis and Immunoblotting

Cells were lysed in lysis buffer containing 50 mM HEPES-NaOH, pH 7.5, 150 mM NaCl, 3 mM MgCl_2_, 1 mM dithiothreitol, 1 mM phenylmethane sulfonylfluoride, 2 mM leupeptin, 1 mM ethylenediaminetetraacetic acid, 1 mM Na_3_VO_4_, 10 mM NaF, and 0.5% NP-40. Lysates were centrifuged to collect supernatants [37]. Unless otherwise indicated, all experiments were carried out at 4 °C. Each sample (25 microgram of total proteins per lane) containing denaturing sample buffer (Fujifilm) was separated using premade sodium dodecylsulfate–polyacrylamide gels (Nacalai Tesque). Proteins were transferred to polyvinylidene fluoride membrane (PVDF; Merck Millipore, Burlington, MA, USA), blocked with Blocking One (Nacalai Tesque), and immunoblotted with primary antibodies followed by peroxidase enzyme-conjugated secondary antibodies. Immunoreactive bands were detected on X-ray film (Fujifilm) or using tetramethylbenzidine (TMB, Nacalai Tesque) and were scanned using CanoScan LiDE 400 (Canon, Tokyo, Japan) and CanoScan LiDE software (ver. OS14, Canon).

Multiple independent experiments were conducted (Figures S9–S17). Band intensities were quantified using Fiji software and normalized to another control sample band. For immunoblotting, the results were derived from three independent experiments (biological replicates), each performed using independently prepared samples.

### 4.5. Affinity-Precipitation Assay for GTP-Bound RhoG (Active RhoG)

Cells were lysed in pull-down buffer containing 50 mM HEPES-NaOH, pH 7.5, 150 mM NaCl, 10 mM MgCl_2_, 1 mM dithiothreitol, 1 mM phenylmethane sulfonylfluoride, 2 mM leupeptin, 3 mM ethylenediaminetetraacetic acid, 1 mM Na_3_VO_4_, 10 mM NaF, and 0.5% NP-40. Lysates were centrifuged to collect supernatants [58]. They were incubated with Protein G-Sepharose 4FF (GE Healthcare, Chicago, IL, USA) preabsorbed with RBD (1 microgram per 500 microgram of total proteins), a region that binds specifically to GTP-bound RhoG (active RhoG), for 30 min at 4 °C with gentle rotation. After incubation, the beads were washed three times with pull-down buffer. The GTP-bound RhoG was subsequently eluted by boiling protein complexes in sample buffer. Finally, both the affinity-precipitated samples and the total lysates were resolved via SDS-PAGE and analyzed by means of immunoblotting using an anti-RhoG antibody.

### 4.6. Detection of Phosphorylated Akt Kinase (Active Akt Kinase)

Cells were lysed using lysis buffer in accordance with the manufacturer’s instructions (Merck). An enzyme-linked immunosorbent assay (ELISA) was then performed to detect active p(Ser473)pan-Akt. Following the sandwich ELISA and the treatment with horseradish peroxidase (HRP)-conjugated antibodies and TMB, the absorbance was measured at 450 nm using an MPR-A100, microplate spectrophotometer (AS ONE Co., Osaka, Japan).

### 4.7. Statistical Analysis

Values are presented as means ± standard deviation (SD) from independent experiments. Intergroup comparisons were performed using unpaired *t*-tests with either Student’s or Welch’s correction in Excel software (ver. 2024, Microsoft, Redmond, WA, USA). For multiple comparisons, one-way analysis of variance (ANOVA) was performed, followed by the Tukey honest significant difference (HSD) test using the StatPlus add-in software (ver. 2021, Alexandria, VA, USA) for Excel. Differences were considered statistically significant at *p* < 0.05. All analyses were performed with investigators blinded to sample conditions.

## 5. Conclusions

Our findings suggest that PTEN signaling contributes to restraining oligodendroglial morphological differentiation and that signaling through RhoG may participate in mediating this regulation. These results provide an in vitro cellular experimental system to examine how inhibitory PTEN activity may be associated with abnormal and/or dysregulated oligodendroglial cell differentiation. While our in vitro findings provide insight into PTEN-dependent regulation of oligodendroglial membrane dynamics, caution is warranted when extrapolating these results to in vivo myelination or human systems, which involve complex developmental and multicellular mechanisms. Future studies using genetic PTEN models and other in vivo systems may be required to determine how these cellular phenotypes translate into white matter morphologies.

## Figures and Tables

**Table 1:** Key materials used in this study.

Reagents or Materials	Companies or Sources	Catalog Number	Concentration Used
Key antibodies			
Anti-2′,3′-cyclic nucleotide phosphodiesterase (CNPase)	Santa Cruz Biotechnology (Santa Cruz, CA, USA)	sc-166019	IB, 1:250
Anti-glutathione S-transferase (GST) pi	MBL (Tokyo, Japan)	312	IB, 1:1000
Anti-actin (also called actin beta type)	MBL	M177-3	IB, 1:10,000
Anti-proteolipid protein 1 (PLP1)	Atlas Antibodies (Stockholm, Sweden)	HPA004128	IB, 1:5000
Anti-claudin-11 (CLDN11, also known as oligodendroglila cell-specific protein)	Abcam (Cambridge, UK)	ab53041	IB, 1:1000
Anti-glyceraldehyde-3-phosphate dehydrogenase (GAPDH)	Santa Cruz Biotechnology	sc-32233	IB, 1:10,000
Anti-mTOR	Santa Cruz Biotechnology	sc-517464	IB, 1:250
Anti-p(Ser1261)mTOR	Sigma-Aldrich (St. Louis, MO, USA)	SAJ560029	IB, 1:250
Anti-Akt	Santa Cruz Biotechnology	sc-5298	IB, 1:50
Anti-p(Thr308)Akt	Santa Cruz Biotechnology	sc-16646	IB, 1:50
Anti-green fluorecence protein (GFP)	Nakalai Tesque (Kyoto, Japan)	GF090R	Immunoprecipitation [IP], 0.2 microgram per 1 microgram of recombinant proteins
Anti-IgG (H+L chain) (Rabbit) pAb-HRP	MBL	458	IB, 1:5000
Anti-IgG (H+L chain) (Mouse) pAb-HRP	MBL	330	IB, 1:5000
Recombinant DNAs			
pcDNA3.1(+)	A control construct was generated from Cat. No. 72035 (Addgene, Watertown, MA, USA)	N/A	1.25 microgram of DNA per 6 cm dish
pcDNA3.1(+)-N-eGFP-RhoG-binding domain (RBD, amino acids 1–81) of human engulfment and cell Motility 1 (ELMO1)	GensScript (Piscataway, NJ, USA)	J196YHL120-6	1.25 microgram of DNA per 6 cm dish

## Data Availability

The datasets used and/or analyzed for the current study are available from the corresponding author upon reasonable request.

## Supplementary Materials

The following supporting information can be downloaded at: https://www.mdpi.com/article/10.3390/ijms27083457/s1.
