# Supplementary material for: The RhoG-Binding Domain of ELMO1 Rescues the PTENopathy-like Phenotype in Oligodendroglial FBD-102b Cells
Source: Int J Mol Sci. 2026 Apr 12;27(8):3457. doi: 10.3390/ijms27083457 (PMC13115920; doi:10.3390/ijms27083457)
Supplement: Supplementary file 1 [file ijms-27-03457-s001.zip › Supplemental figure legends.pdf]

Supplemental figure legends

Figure S1. Treatment with bpV(HOpic) or VO-OHpic induces excessive morphological differentiation. (A) FBD-102b cells were differentiated in the presence of control vehicle for the indicated durations. Quantification of differentiated cells is shown (**, $p < 0.01$; n = 10 fields). (B) Cells were differentiated for several days in the presence of bpV(HOpic). The percentages of differentiated cells are quantified (**, $p < 0.01$; n = 10 fields). (C) Cells were differentiated for several days in the presence of VO-OHpic. The percentages of differentiated cells are quantified (**, $p < 0.01$; n = 10 fields).

Figure S2. Treatment with bpV(HOpic) or VO-OHpic promotes the phosphorylation of mTOR and Akt, thereby increasing their activity downstream of PTEN inhibition. (A-D) FBD-102b cells were cultured in the presence of control vehicle or each inhibitor. Cell lysates were subjected to immunoblotting using an antibody against p(Ser1261)mTOR, mTOR, p(Thr308)Akt, or Akt. Quantification of each immunoreactive band intensity normalized to the total protein (mTOR or Akt) is shown (**, $p < 0.01$; n = 3 blots).

Figure S3. Treatment with bpV(HOpic) or VO-OHpic induces excessive morphological differentiation, which is attenuated by RBD or curcumin. (A-F) FBD-102b cells were differentiated in the presence or absence (vehicle) of the indicated inhibitors, with or without transfection of RBD or the control vector. Individual cell areas are plotted (**, p < 0.01; n = 100 cells from 3 independent dishes prepared in separate experimental batches).

Figure S4. Treatment with bpV(HOpic) or VO-OHpic upregulates differentiation marker proteins. (A-D) FBD-102b cells were treated with the respective inhibitors. Cell lysates prepared 2 days after induction of differentiation were subjected to immunoblotting using antibodies against PLP1, CLDN11, and control GAPDH proteins. Quantification of each immunoreactive band intensity normalized to the loading control is shown (**, $p < 0.01$; n = 3 blots).

Figure S5. RBD recovers the expression levels of differentiation marker proteins. (A-D) FBD-102b cells were transfected with the plasmid coding RBD (+) or its vector (-) and treated in the presence of the respective inhibitors. Cell lysates prepared 2 days after induction of differentiation were subjected to immunoblotting using antibodies against PLP1, CLDN11, and control GAPDH proteins. Quantification of each immunoreactive band intensity normalized to the loading control is shown (**, $p < 0.01$; n = 3 blots).

Figure S6. Treatment with bpV(HOpic) or VO-OHpic increases GTP-bound form of RhoG (A-D) FBD-102b cells were cultured in the presence of control vehicle or each inhibitor. Cell lysates were subjected to affinity-precipitation assay to detect GTP-bound RhoG (**, $p < 0.01$; n = 3 blots).

Figure S7. Curcumin recovers the expression levels of differentiation marker proteins. (A-D) FBD-102b cells were treated with curcumin (+) or vehicle (-), together with the

respective inhibitors. Cell lysates prepared 2 days after induction of differentiation were subjected to immunoblotting using antibodies against PLP1, CLDN11, and control GAPDH proteins. Quantification of each immunoreactive band intensity normalized to the loading control is shown (**, $p < 0.01$; n = 3 blots).

Figure S8. Curcumin recovers the expression levels of differentiation marker proteins. (A-D) FBD-102b cells were treated with MK-2206 (+) or vehicle (-), together with the respective inhibitors. Cell lysates prepared 2 days after induction of differentiation were subjected to immunoblotting using antibodies against PLP1, CLDN11, and control GAPDH proteins. Quantification of each immunoreactive band intensity normalized to the loading control is shown (**, $p < 0.01$; n = 3 blots).

Figure S9. Computer-saved full-sized, unprocessed blot images for Figures 1 and 2. The areas corresponding to the cropped images in the figures are outlined.

Figure S10. Computer-saved full-sized, unprocessed blot images for Figures 3 and 4. The areas corresponding to the cropped images in the figures are outlined.

Figure S11. Computer-saved full-sized, unprocessed blot images for Figures 5 and 6. The areas corresponding to the cropped images in the figures are outlined.

Figure S12. Computer-saved full-sized, unprocessed blot images for Figures S2. The areas corresponding to the cropped images in the figures are outlined.

Figure S13. Computer-saved full-sized, unprocessed blot images for Figures S4. The areas corresponding to the cropped images in the figures are outlined.

Figure S14. Computer-saved full-sized, unprocessed blot images for Figures S5. The areas corresponding to the cropped images in the figures are outlined.

Figure S15. Computer-saved full-sized, unprocessed blot images for Figures S6. The areas corresponding to the cropped images in the figures are outlined.

Figure S16. Computer-saved full-sized, unprocessed blot images for Figures S7. The areas corresponding to the cropped images in the figures are outlined.

Figure S17. Computer-saved full-sized, unprocessed blot images for Figures S8. The areas corresponding to the cropped images in the figures are outlined.
