# Supplementary figures and images for: The RhoG-Binding Domain of ELMO1 Rescues the PTENopathy-like Phenotype in Oligodendroglial FBD-102b Cells
Source: Int J Mol Sci. 2026 Apr 12;27(8):3457. doi: 10.3390/ijms27083457 (PMC13115920; doi:10.3390/ijms27083457)

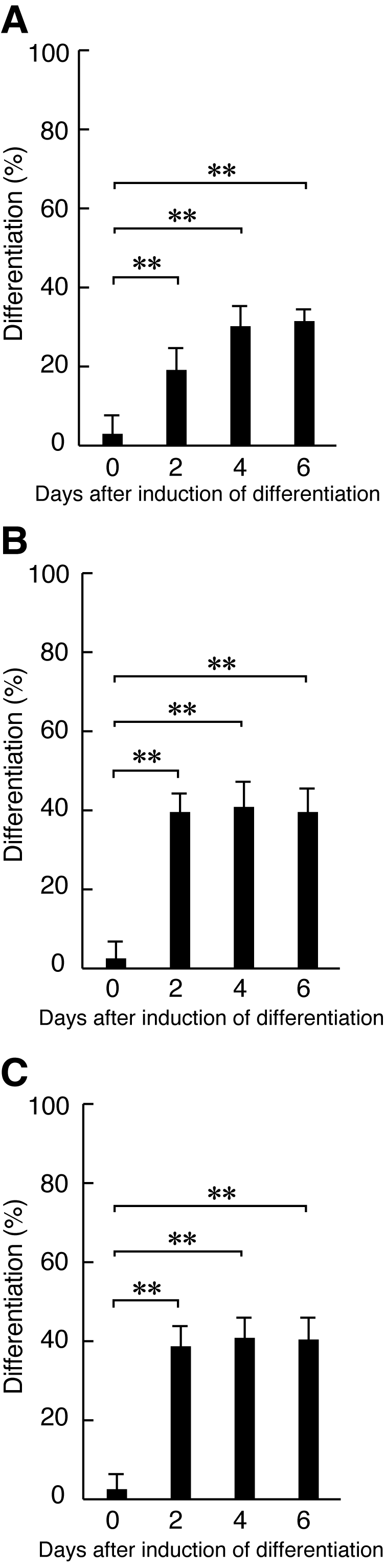

Supplement: Supplementary file 1 [file ijms-27-03457-s001.zip › Figure S1.tif]

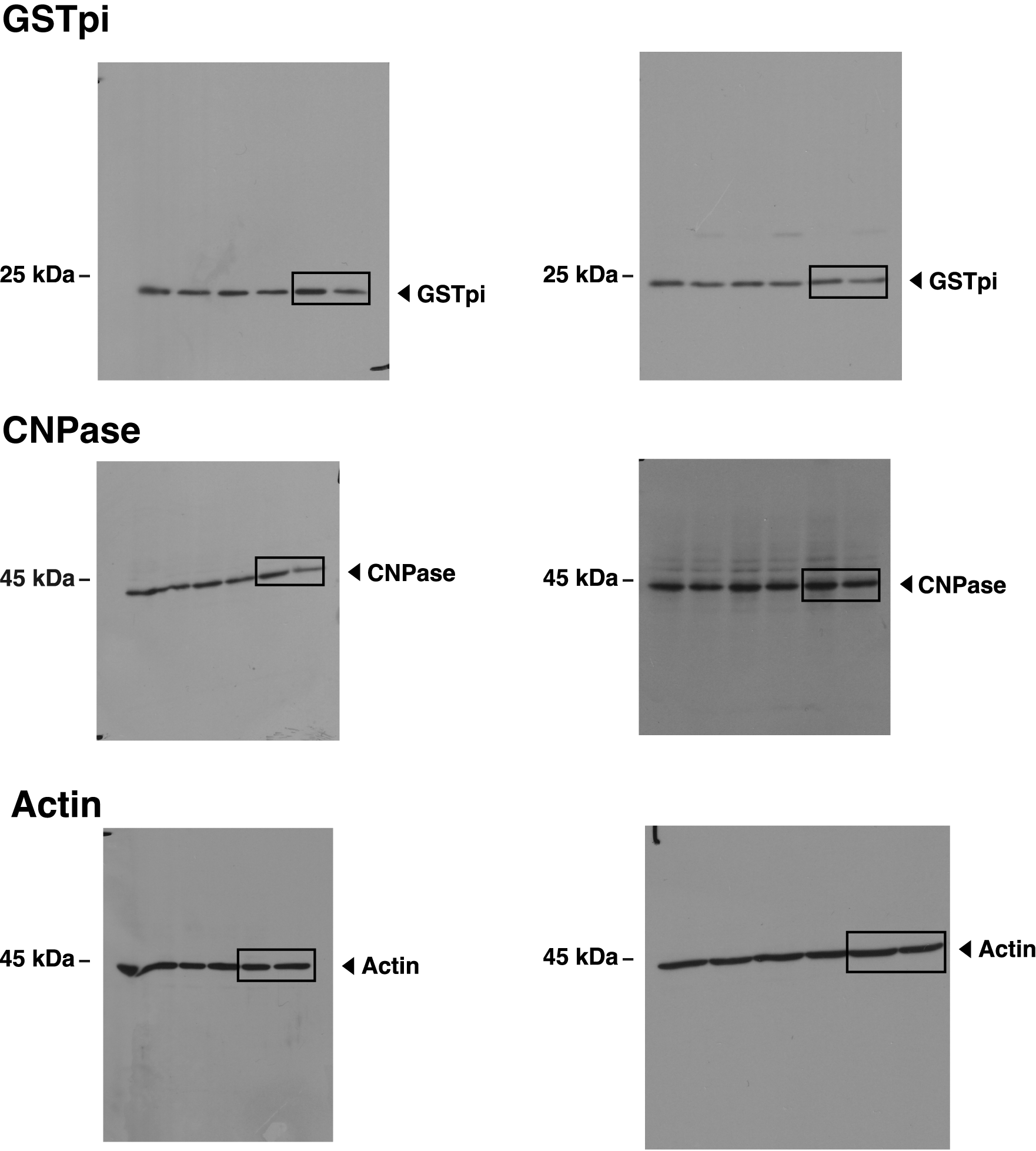

Supplement: Supplementary file 1 [file ijms-27-03457-s001.zip › Figure S10.Full size gel for Figures 3 and 4.tif]

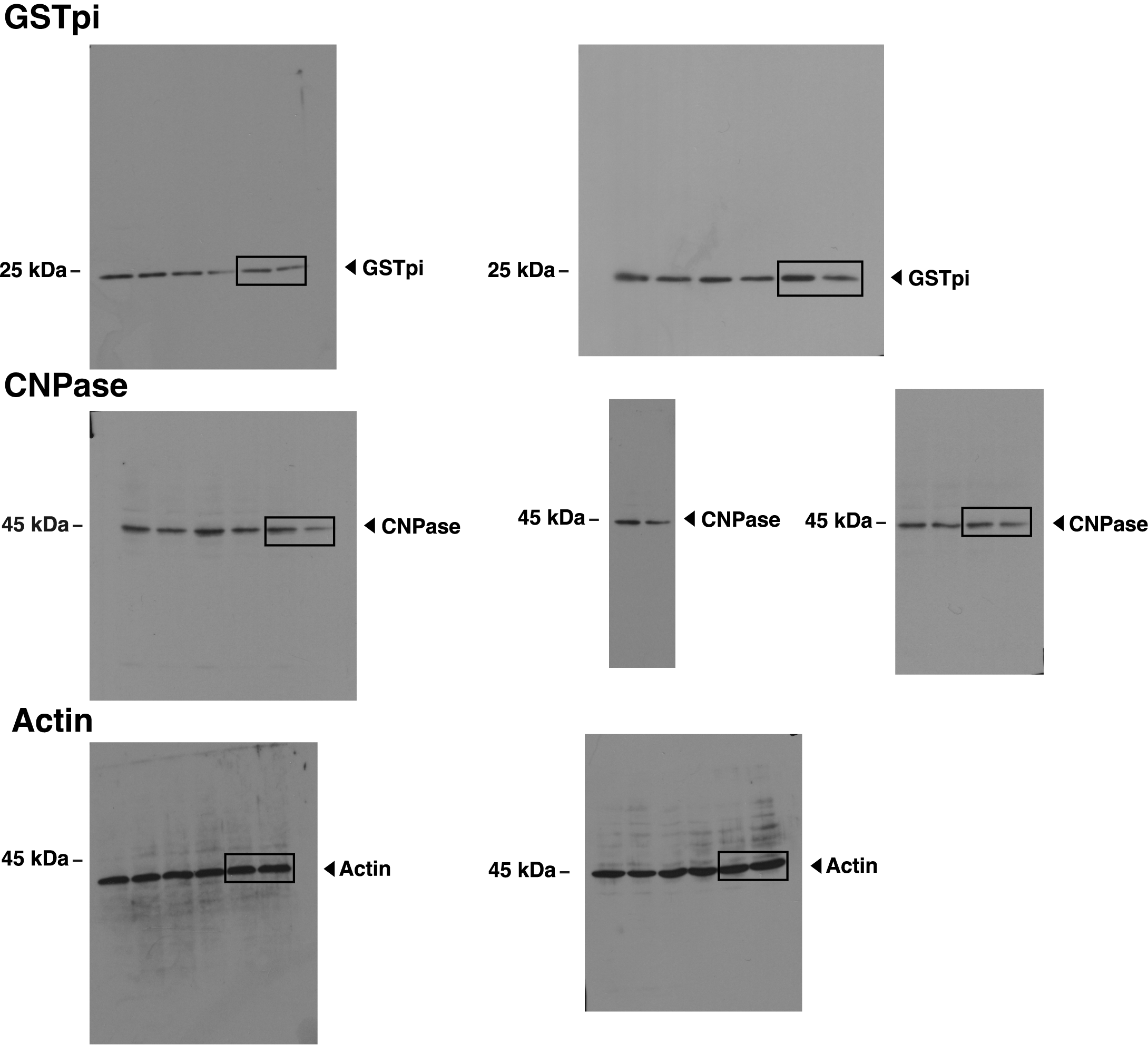

Supplement: Supplementary file 1 [file ijms-27-03457-s001.zip › Figure S11.Full size gel for Figures 5 and 6.tif]

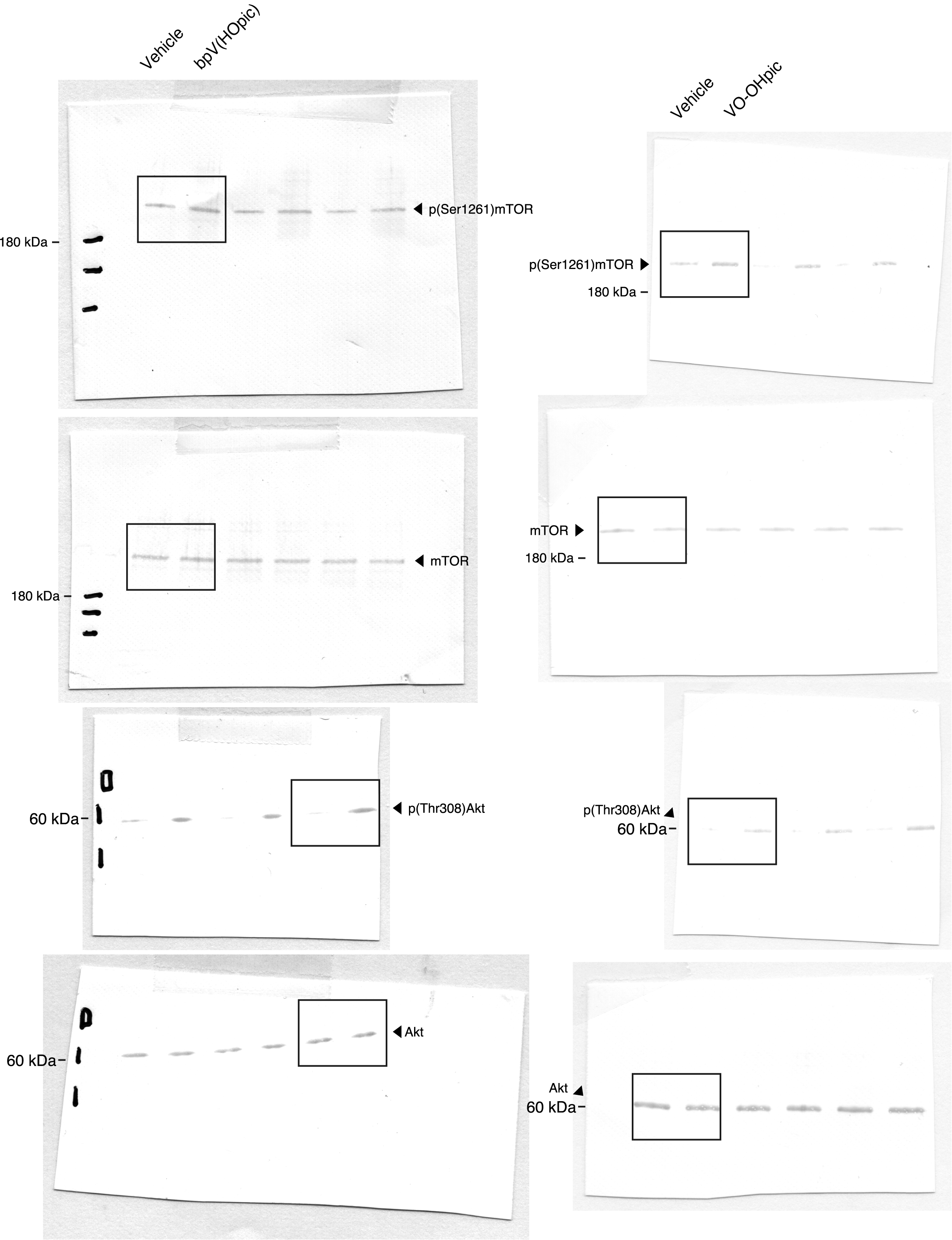

Supplement: Supplementary file 1 [file ijms-27-03457-s001.zip › Figure S12.Full size gel for Figure S2.tif]

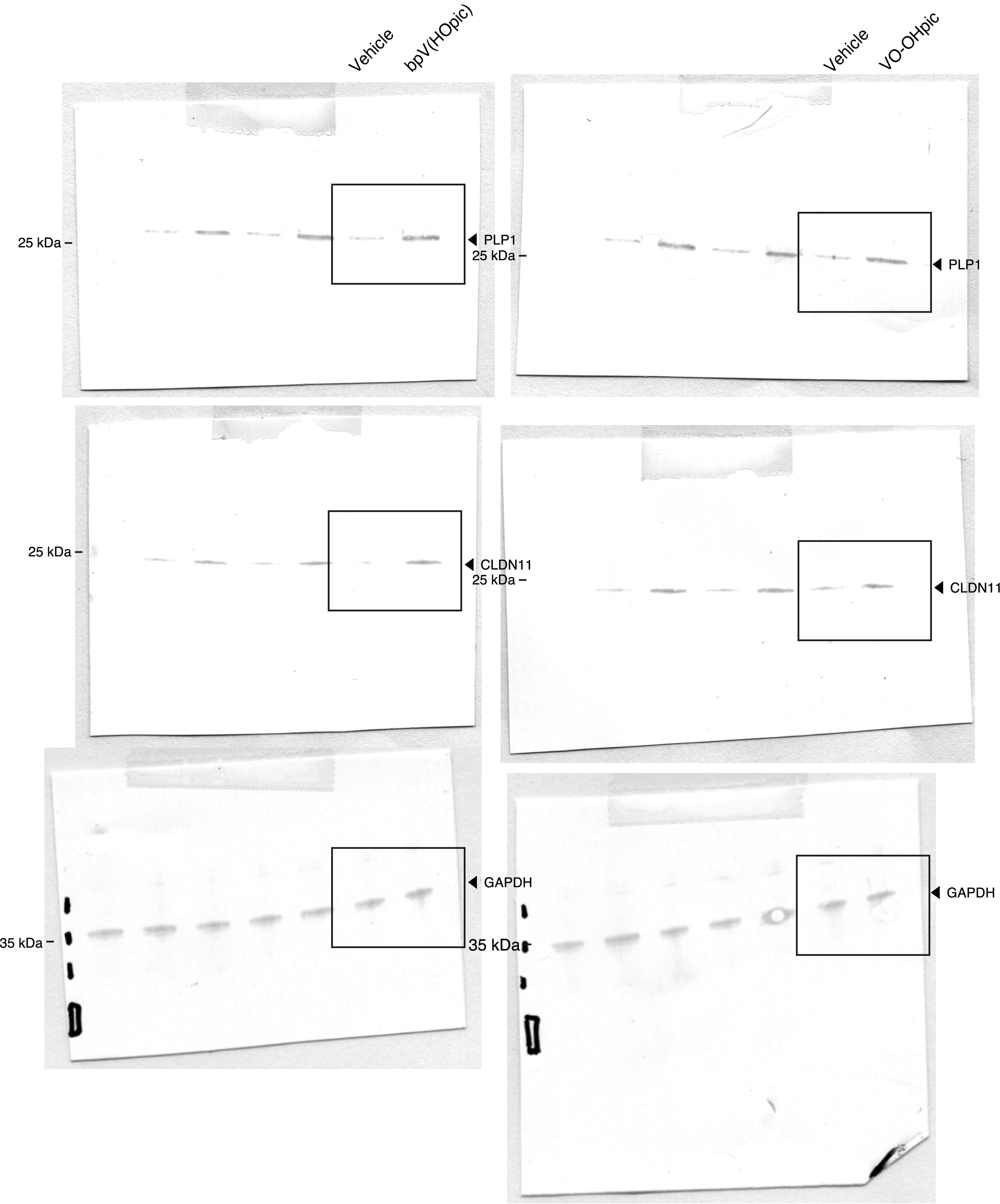

Supplement: Supplementary file 1 [file ijms-27-03457-s001.zip › Figure S13.Full size gel for Figure S4.tif]

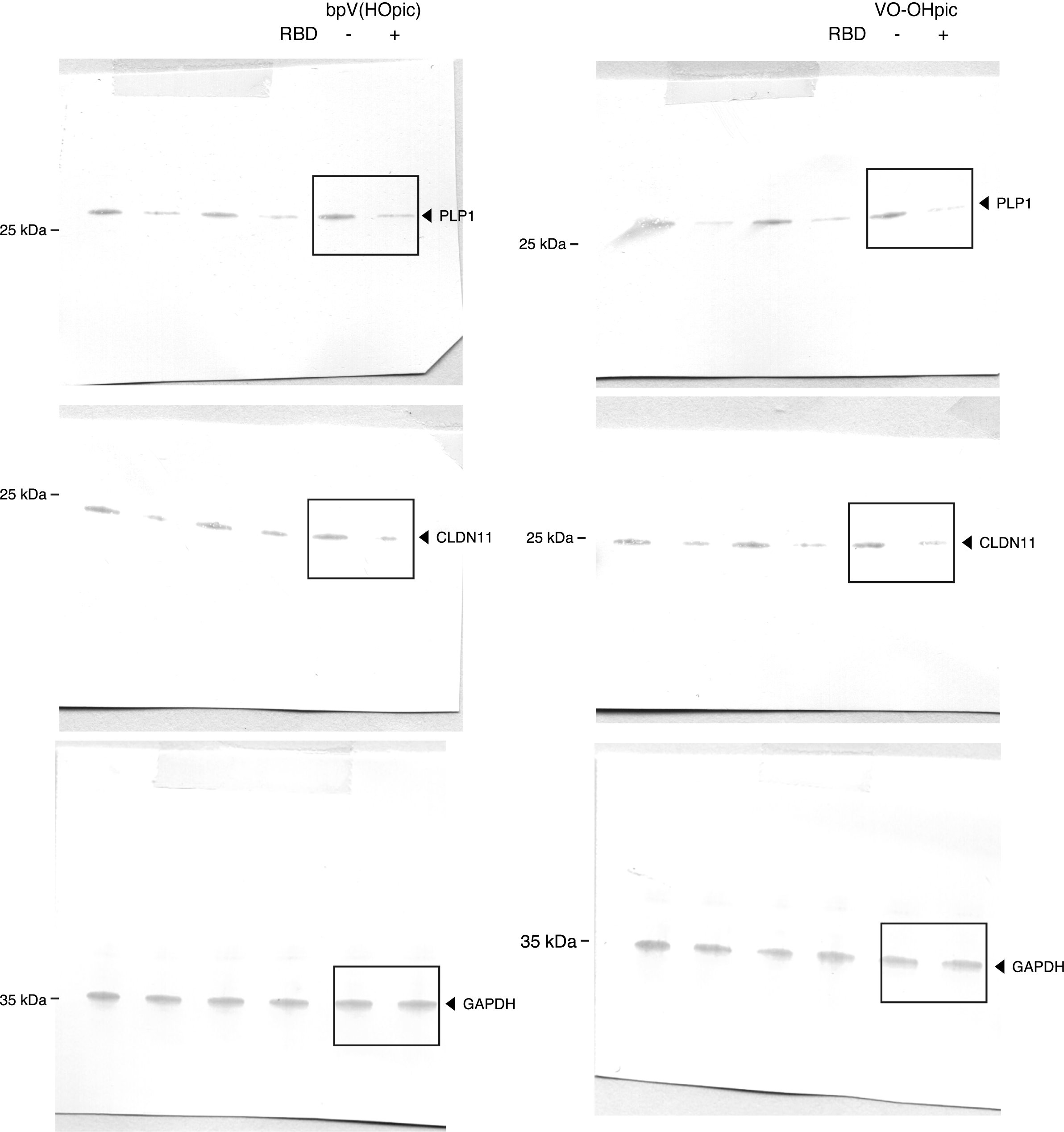

Supplement: Supplementary file 1 [file ijms-27-03457-s001.zip › Figure S14.Full size gel for Figure S5.tif]

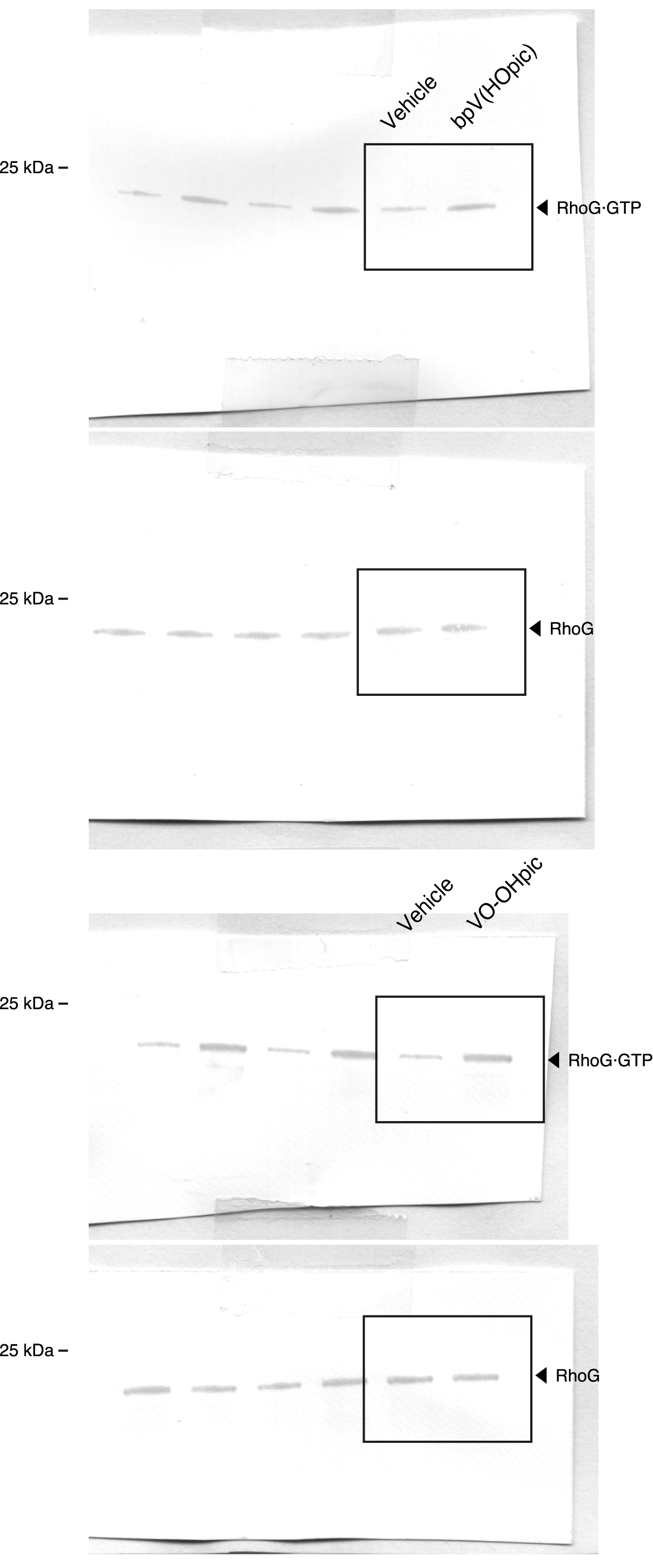

Supplement: Supplementary file 1 [file ijms-27-03457-s001.zip › Figure S15.Full size gel for Figure S6.tif]

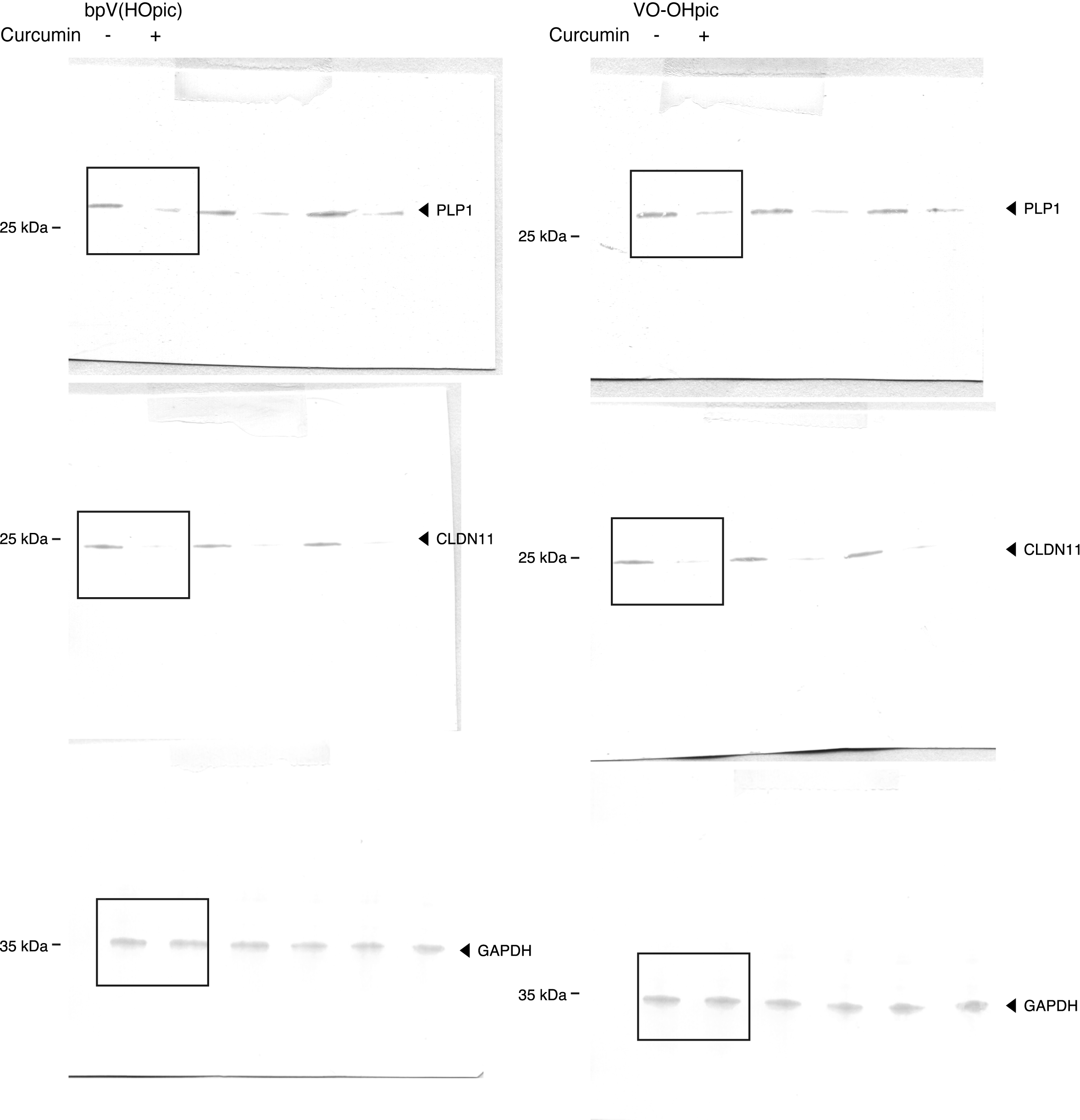

Supplement: Supplementary file 1 [file ijms-27-03457-s001.zip › Figure S16.Full size gel for Figure S7.tif]

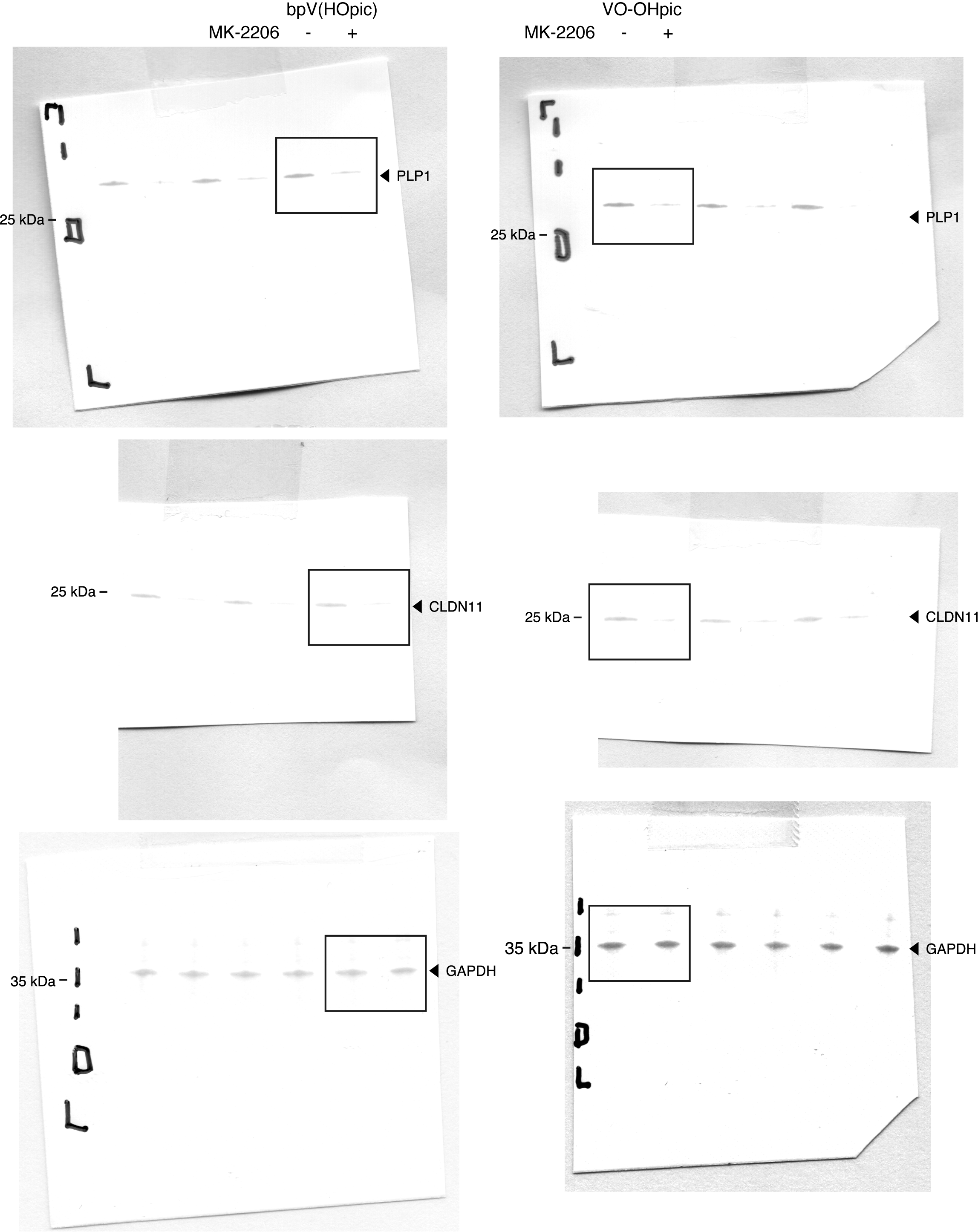

Supplement: Supplementary file 1 [file ijms-27-03457-s001.zip › Figure S17.Full size gel for Figure S8.tif]

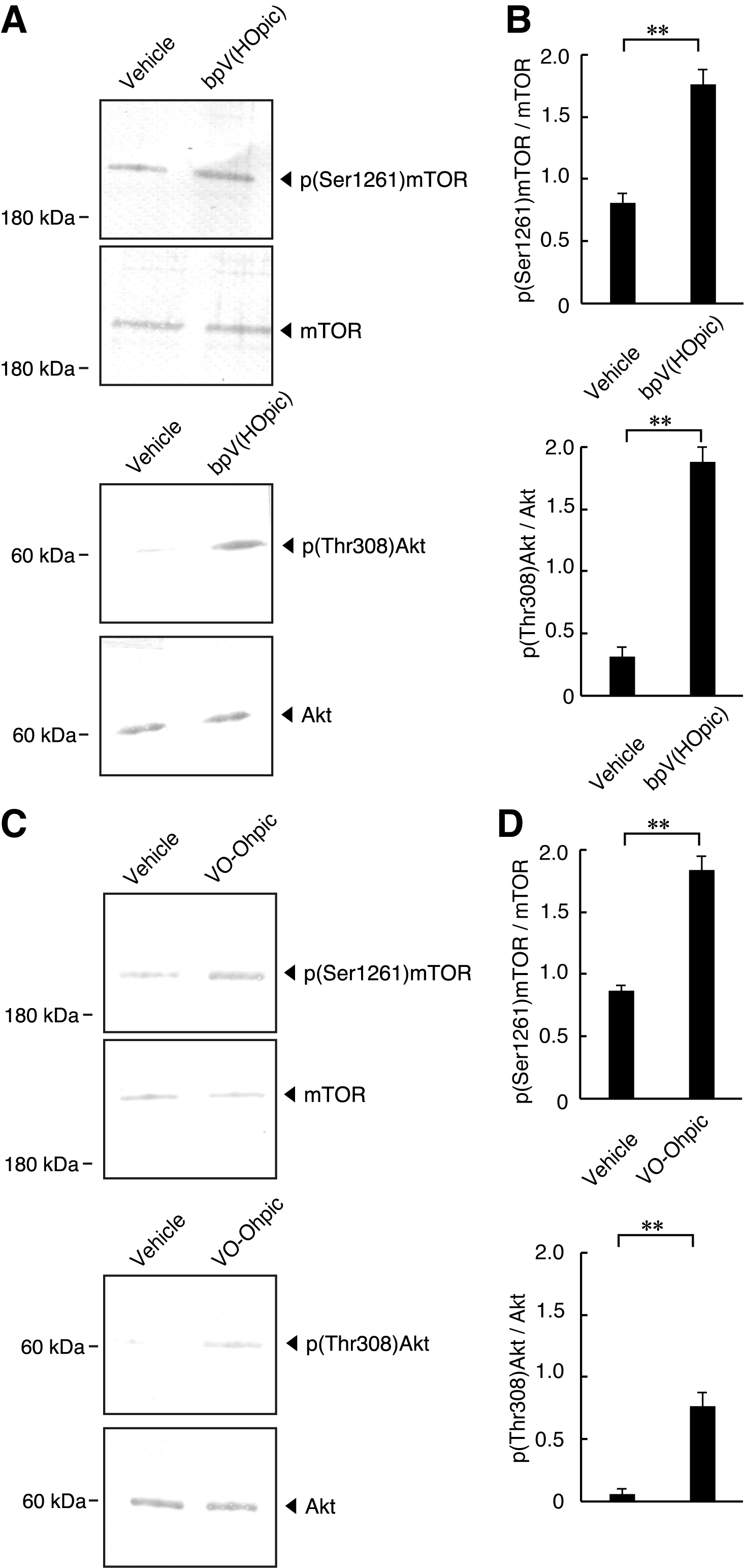

Supplement: Supplementary file 1 [file ijms-27-03457-s001.zip › Figure S2.tif]

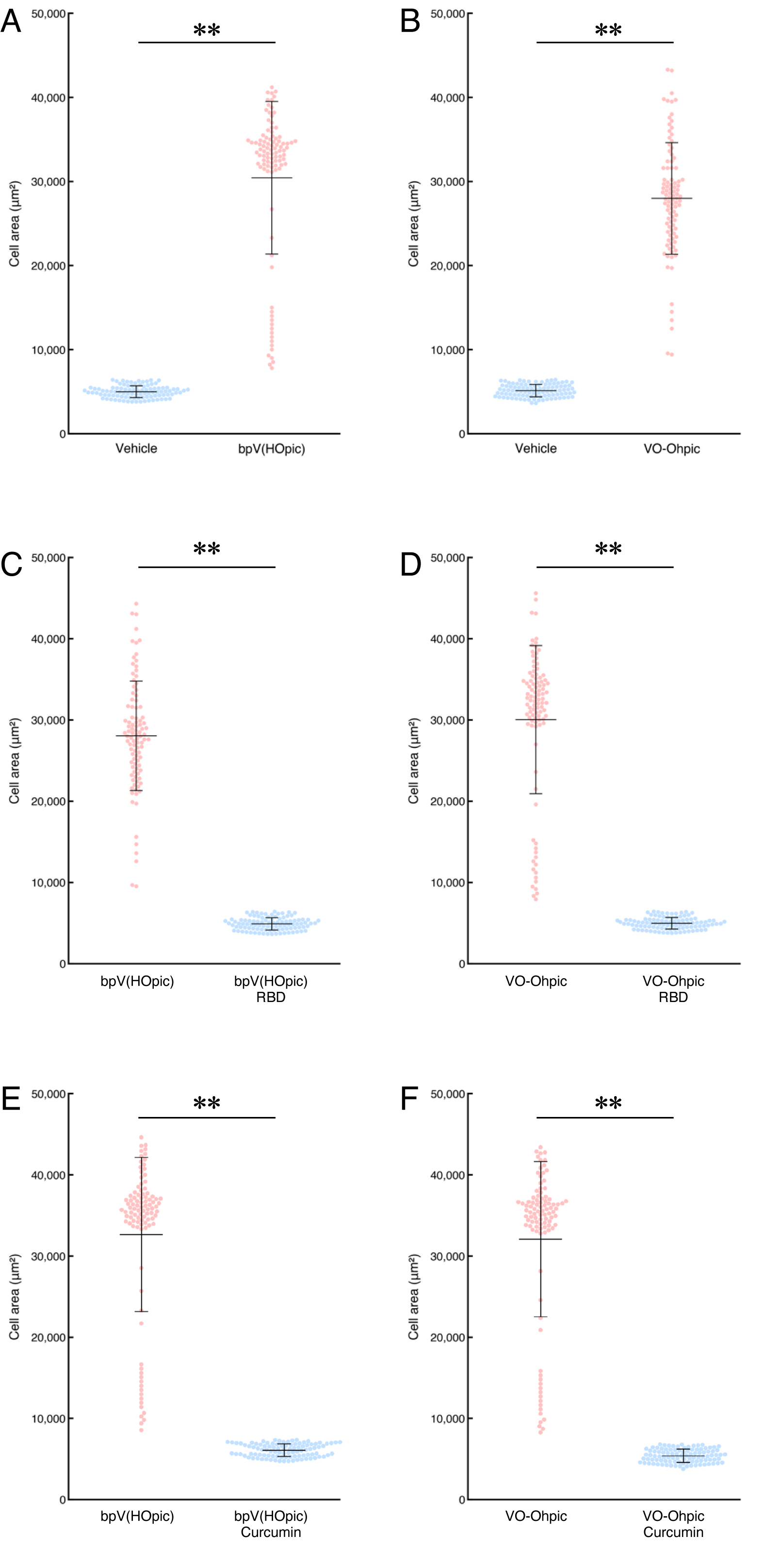

Supplement: Supplementary file 1 [file ijms-27-03457-s001.zip › Figure S3.tif]

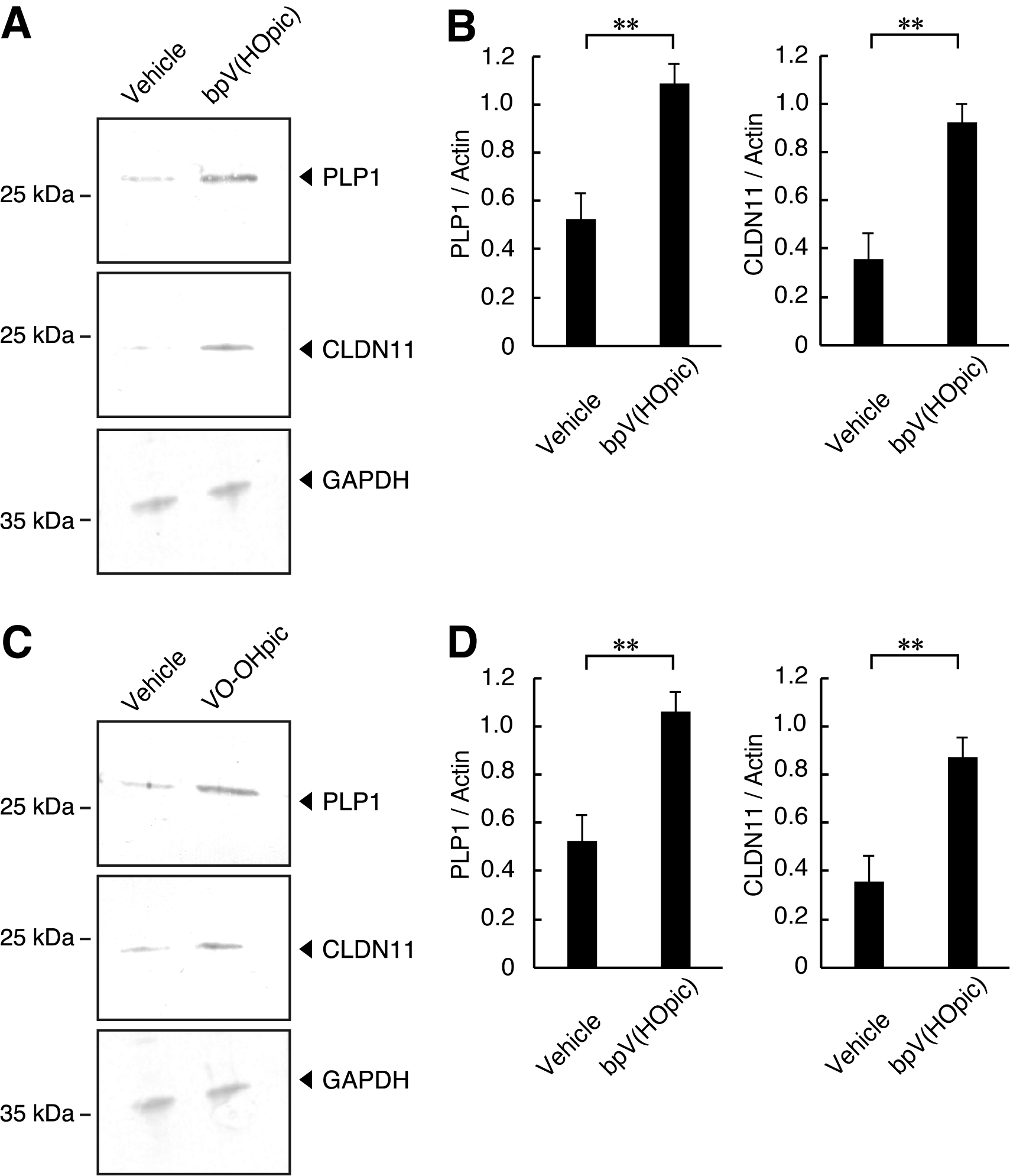

Supplement: Supplementary file 1 [file ijms-27-03457-s001.zip › Figure S4.tif]

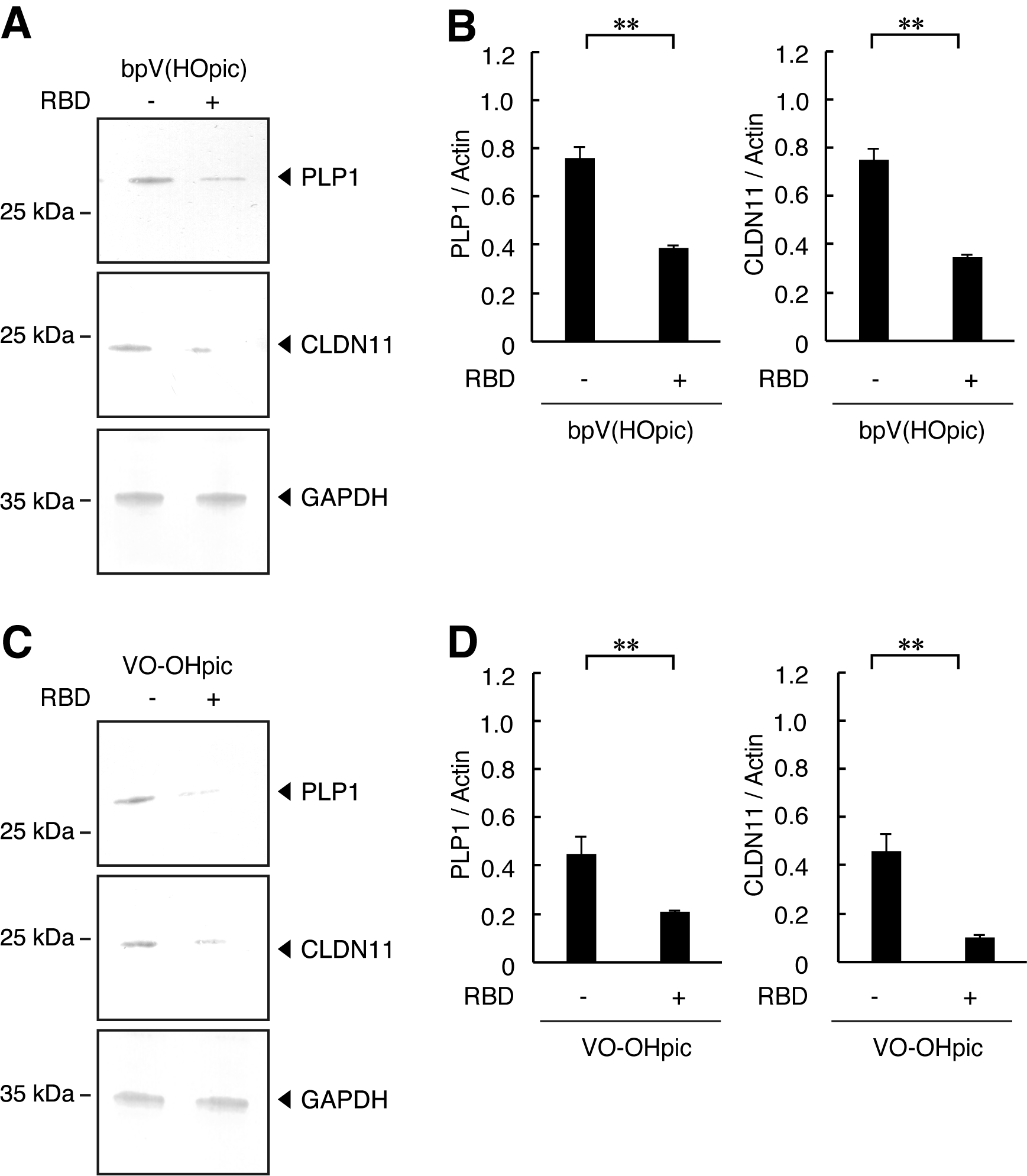

Supplement: Supplementary file 1 [file ijms-27-03457-s001.zip › Figure S5.tif]

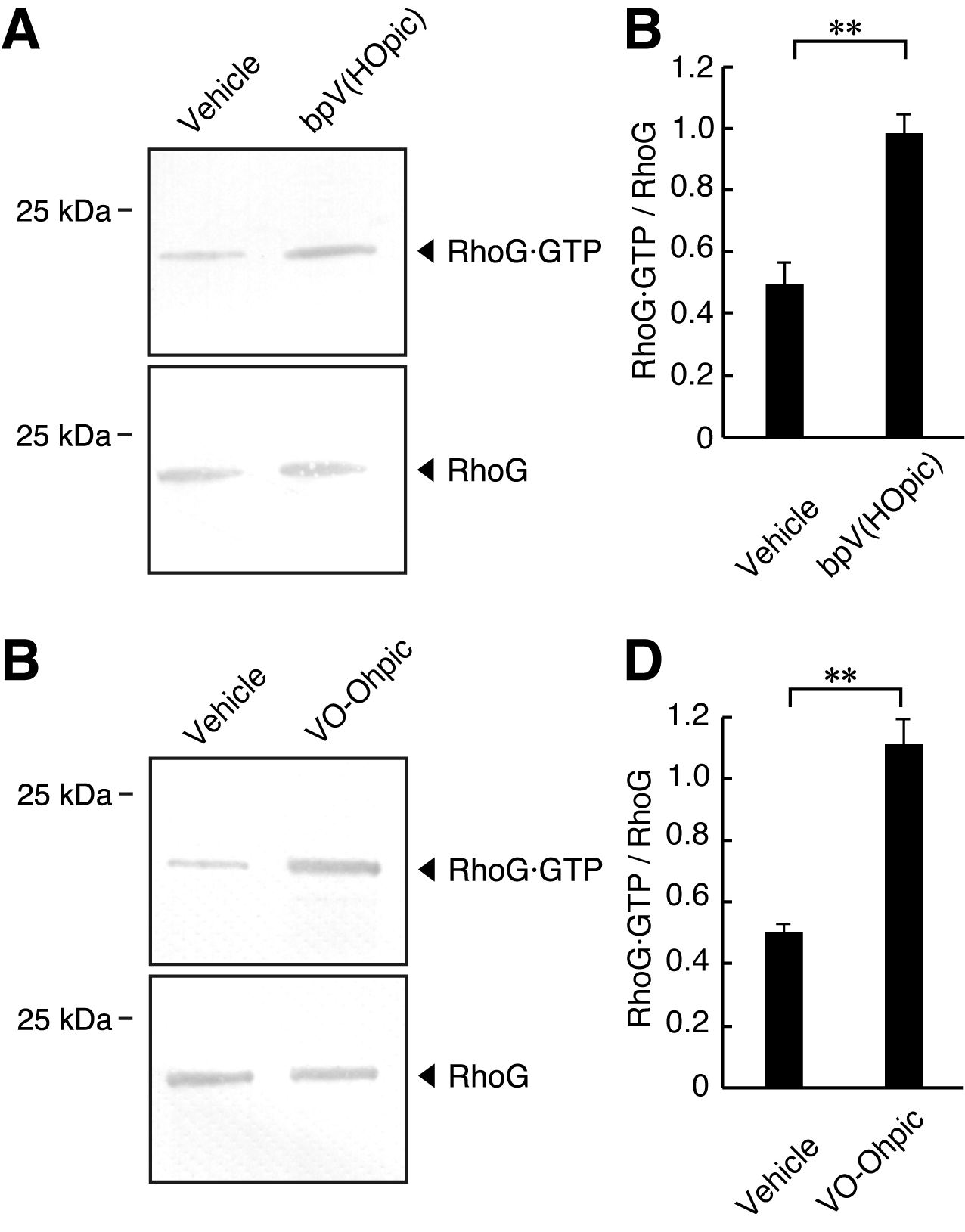

Supplement: Supplementary file 1 [file ijms-27-03457-s001.zip › Figure S6.tif]

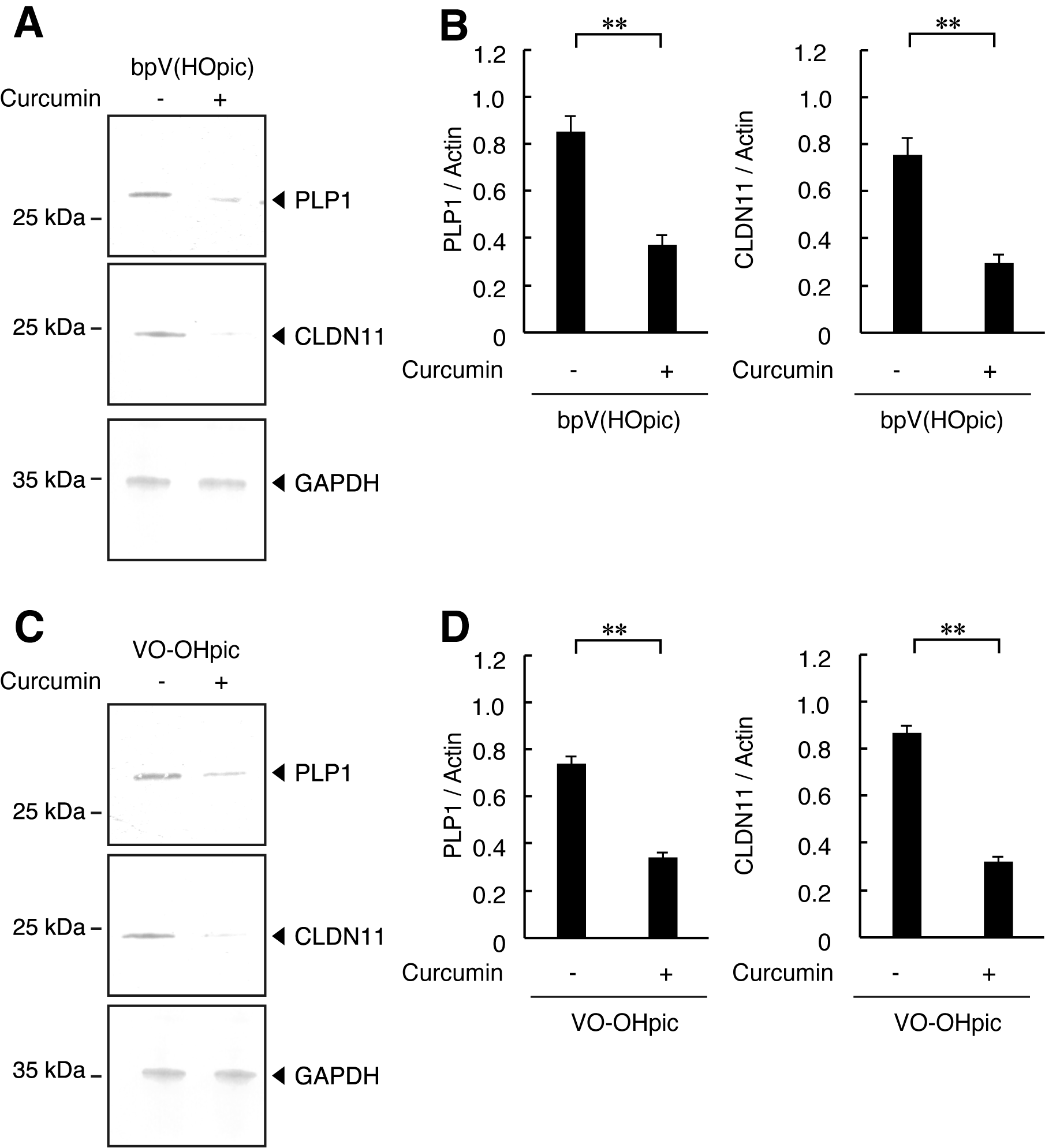

Supplement: Supplementary file 1 [file ijms-27-03457-s001.zip › Figure S7.tif]

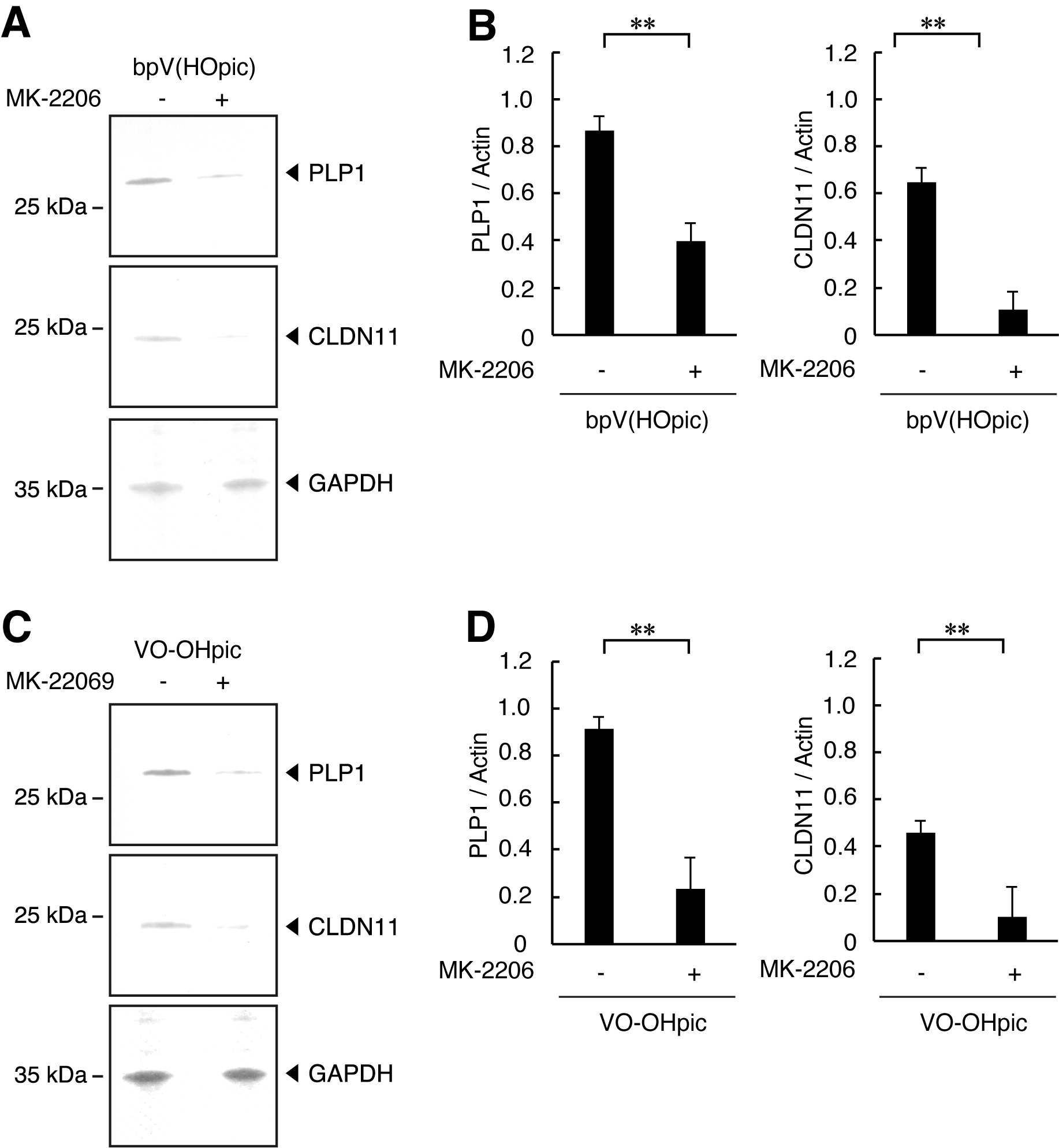

Supplement: Supplementary file 1 [file ijms-27-03457-s001.zip › Figure S8.tif]

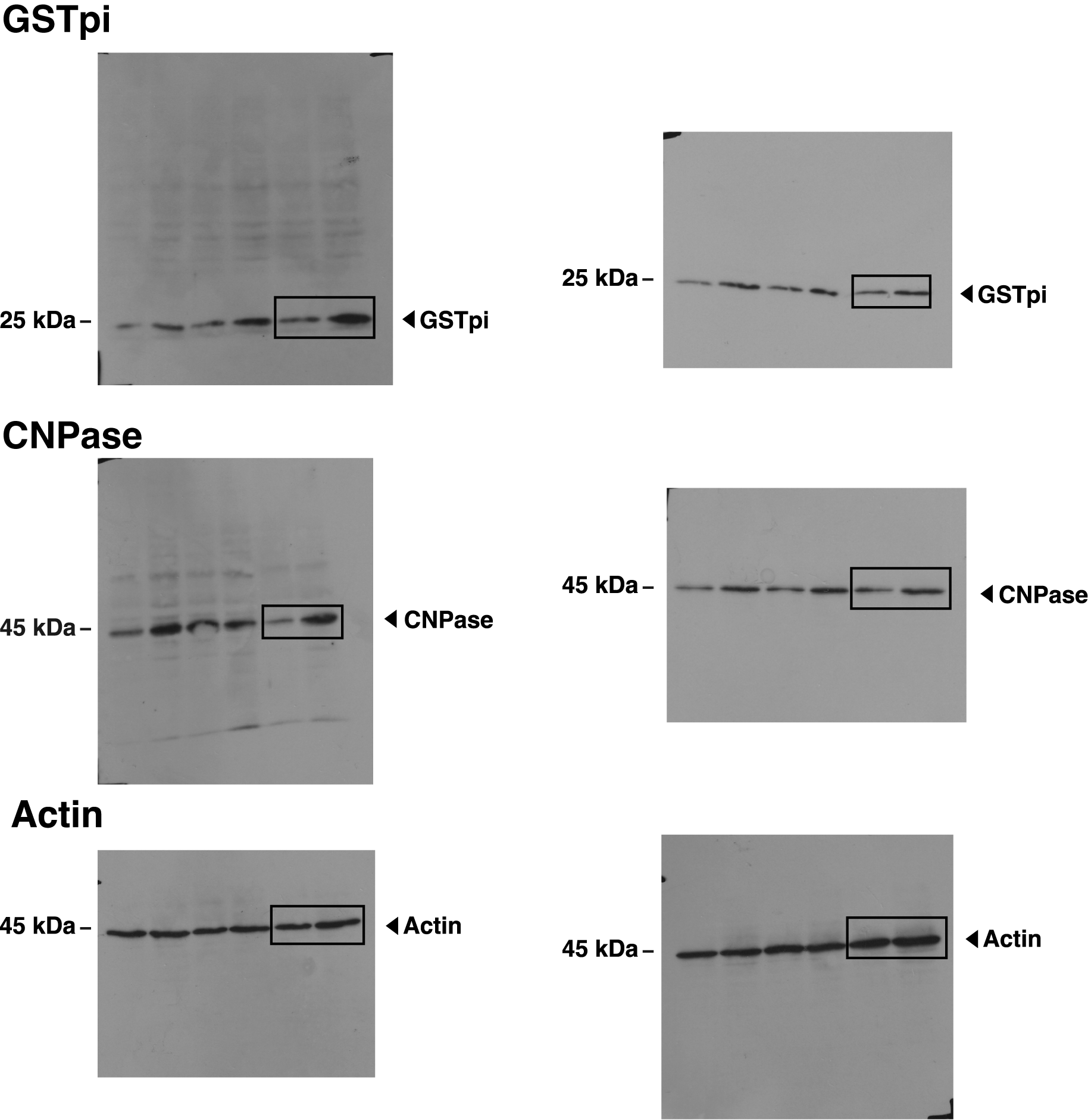

Supplement: Supplementary file 1 [file ijms-27-03457-s001.zip › Figure S9.Full size gel for Figures 1 and 2.tif]
